# Potential Efficacy of *β*-Amyrin Targeting Mycobacterial Universal Stress Protein by In Vitro and In Silico Approach

**DOI:** 10.3390/molecules27144581

**Published:** 2022-07-18

**Authors:** Md Amjad Beg, Shivangi, Obaid Afzal, Md Sayeed Akhtar, Abdulmalik S. A. Altamimi, Afzal Hussain, Md Ali Imam, Mohammad Naiyaz Ahmad, Sidharth Chopra, Fareeda Athar

**Affiliations:** 1 Centre for Interdisciplinary Research in Basic Science, Jamia Millia Islamia, Jamia Nagar, New Delhi 110025, Uttar Pradesh, India; md164429@st.jmi.ac.in (M.A.B.); ali.imamuit@gmail.com (M.A.I.); 2 CSIR-Institute of Genomics and Integrative Biology, Mall Road, Delhi 110007, Uttar Pradesh, India; mathuria93shivangi@gmail.com; 3 Department of Pharmaceutical Chemistry, College of Pharmacy, Prince Sattam Bin Abdulaziz University, Al-Kharj 11942, Saudi Arabia; as.altamimi@psau.edu.sa; 4 Department of Clinical Pharmacy, College of Pharmacy, King Khalid University, Abha 61421, Saudi Arabia; mdhusain@kku.edu.sa; 5 Department of Pharmaceutics, College of Pharmacy, King Saud University, Riyadh 11451, Saudi Arabia; afzal.pharma@gmail.com; 6 Division of Molecular Microbiology and Immunology, CSIR-Central Drug Research Institute, Sector 10, Janakipuram Extension, Sitapur Road, Lucknow 226031, Uttar Pradesh, India; naiyaz.ahmad@gmail.com (M.N.A.); skchopra007@gmail.com (S.C.); 7 Academy of Scientific and Innovative Research (AcSIR), Ghaziabad 201002, Uttar Pradesh, India

**Keywords:** *A. aspera*, *β*-amyrin, *C. gigantea*, *C. procera*, GC-MS, Minimum inhibitory concentration (MIC), Molecular docking, MD simulations, *Tuberculosis* (TB)

## Abstract

The emergence of drug resistance and the limited number of approved antitubercular drugs prompted identification and development of new antitubercular compounds to cure *Tuberculosis* (TB). In this work, an attempt was made to identify potential natural compounds that target mycobacterial proteins. Three plant extracts (*A. aspera*, *C. gigantea* and *C. procera*) were investigated. The ethyl acetate fraction of the aerial part of *A. aspera* and the flower ash of *C. gigantea* were found to be effective against *M. tuberculosis* H_37_Rv. Furthermore, the GC-MS analysis of the plant fractions confirmed the presence of active compounds in the extracts. The *Mycobacterium* target proteins, i.e., available PDB dataset proteins and proteins classified in virulence, detoxification, and adaptation, were investigated. A total of ten target proteins were shortlisted for further study, identified as follows: *BpoC*, *RipA*, *MazF4*, *RipD*, *TB15.3*, *VapC15*, *VapC20*, *VapC21*, *TB31.7*, and *MazF9*. Molecular docking studies showed that *β*-amyrin interacted with most of these proteins and its highest binding affinity was observed with *Mycobacterium* Rv1636 (*TB15.3*) protein. The stability of the protein-ligand complex was assessed by molecular dynamic simulation, which confirmed that *β*-amyrin most firmly interacted with *Rv1636* protein. *Rv1636* is a universal stress protein, which regulates *Mycobacterium* growth in different stress conditions and, thus, targeting *Rv1636* makes *M. tuberculosis* vulnerable to host-derived stress conditions.

## 1. Introduction

*Tuberculosis* (TB) is primarily caused by *Mycobacterium tuberculosis* (*M. tuberculosis*) and considered to be an airborne disease that spreads through sneezing (air borne fine droplets), direct contact, and sharing personal daily use items [1]. Many attempts have been made to control and cure TB and its related critical consequences. However, several factors complicate the treatment strategy, such as the emergence of multidrug resistance against established drugs due to regular mutations, patients having poor access to drugs, long-term therapy, poor patient adherence, and severe dose-dependent side effects [2]. These factors have resulted in progressive growth of latent and active TB cases annually, as reported by the World Health Organization in 2021 (WHO, 2021). The WHO estimated that there were approximately 9.9 million TB cases in 2020, wherein there were about 1.3 million with HIV-negative and 0.214 million with HIV-positive cases [3,4,5]. Notably, the disease affects patients of all ages, the elderly, and the immunocompromised [6,7]. Circulating TB strains are now resistant to a variety of therapeutic combinations and because the discovery of novel drugs takes time, an alternative approach to provide adjuvants that can boost antibiotics potency is to be considered. It was reported that bacterial susceptibility to antibiotics increases with the co-administration of some natural products [8,9,10].

In this study, we investigated three medicinal plants, *Achyranthes aspera* (*A. aspera*), *Calotropis gigantea* (*C. gigantea*) and *Calotropis procera* (*C. procera*), for their potential therapeutic intervention in TB. *A. aspera* is a widely known weed in many southeast Asian countries. The plant has been used as a folk medicine in Australia, Kenya, and India, since ancient times. In India, it has been used to treat dropsy, hydrophobia, snake bites, ophthalmic, and cutaneous diseases [11,12,13]. As a part of the Amaranthaceous family, it is also used to treat asthma, kidney stones, skin diseases, and epilepsy [14]. Previous studies have also reported it as being antidepressant, antioxidant, anxiolytic, anticonvulsant, antihyperglycemic, antiallergenic, anti-obese, hypolipidemic, and hepatoprotective [15,16,17,18,19].

*Calotropis* is a common wasteland weed, commonly known as milkweed or swallowwort [20]. It is a member of the Asclepiadaceae family (Milkweeds), which has almost 2000 species globally and has a common place in the tropics, and subtropics, but is rare in cold climates [21]. Traditionally, *Calotropis* has been used to cure common ailments, such as cold, asthma, vomiting, and diarrhea. The dried whole plant is a tonic, expectorant, depurative, and anthelmintic, according to Ayurveda. Asthma, bronchitis, and dyspepsia are treated with the powdered root. Paralysis, arthralgia, swellings, and intermittent fevers can be treated with the leaves and its flowers, due to its bitter, digestive, astringent, stomachic, anthelmintic, and tonic properties. Moreover, it is a well-known homoeopathic remedy [22]. 

*C. procera* is an Asclepiadaceae shrub native to Egypt. It has purgative, antibacterial, anthelmintic, anticoagulant, antipyretic, anti-inflammatory, analgesic, and neuromuscular blocking properties [23,24,25,26,27]. The plant’s extract has physiological effects on cardiac, soft, and skeletal muscular tissues. In traditional medicine, the genus *Calotropis* is used to treat leprosy, ulcers, tumors, liver, and piles problems. It also has potential anticancer effects [28]. The key phytoconstituents are practically found in every *Calotropis* species. However, the relative distribution in individual plants can vary depending upon environmental conditions [29,30,31,32].

The increased incidence of TB cases and drug resistant strains prompted us to identify novel drug targets and natural therapeutics with lesser toxicity. Considering the broad spectrum of biological properties of *A. aspera*, *C. gigantea* and *C. procera* medicinal plants, we investigated the effect of their phytoconstituents on mycobacterial proteins and their survival.

## 2. Results

### 2.1. Identification and Procurement of the Plant Materials

The aerial and root parts of *A. aspera*, and flowers of *C. gigantea* and *C. procera* were collected from the burial ground in Shahjahanpur, Uttar Pradesh. They were identified by their flower and inflorescence. *C. gigantea* and *C. procera* contain white and purple flowers, respectively, as the major phenotypic differentiation [33,34].

### 2.2. Preparation of Plant Extracts

The plant was dried in shade followed by cutting into small pieces and grinding into fine powder. The dried powdered material of aerial and root parts were 1500 g and 300 g, respectively. The dried powdered material of the flower parts of *C. procera, C. gigantea* and *C. gigantea ash* were 200 g each. The flower ash was obtained by burning the flowers in petroleum ether. The powder of *A. aspera* was soaked in methanol (10 times *w*/*v*) and the powders of *C. procera* and *C. gigantea* were soaked in methanol (5 times *w*/*v*), at room temperature, for 10 days and 5 days, respectively, depending upon the weight of the powders. The extracted content was first filtered by Whatman filter paper (150 mm) and then evaporated using a Heidolph rotary evaporator. The crude methanol extract was successively fractionated in various solvents, i.e., hexane, ethyl acetate, ethanol and water, in order of their increasing polarity [35,36]. Total yield of the extractable components (EC) from *A. aspera* (aerial and root parts), *C. procera* (flower), *C. gigantea* (flower) and *C. gigantea* (flower ash) in various solvents are shown in Table 1.

### 2.3. Phytochemical Screening of Plant Extracts

Phytochemical tests of each extract fraction were performed as mentioned in the method section for alkaloids, tannins, saponins, terpenoids, and organic acids. The phytochemical screening of *A. aspera*, *C. procera* and *C. gigantea* plant extracts in various fractions showed the presence of alkaloids, tannins, saponins, terpenoids, and organic acid, as reported in literature [37,38] (Table 2).

### 2.4. Detection of Total Flavonoid Content (TFC) of Plants

**Figure 1:**
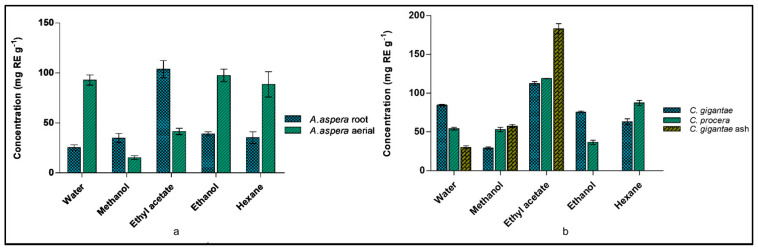
Total flavonoid content determination (**a**) *A. aspera* (**b**) *C. gigantea* and *C. procera*.

Flavonoids are secondary metabolites having multiple physiological activities in plant development, pigmentation, and UV protection, and in defense and signaling pathways between plants and microbes [39,40]. The total flavonoid contents of *A. aspera* (aerial and root), *C. procera*, *C. gigantea* and *C. gigantea ash* parts were analyzed in water, methanol, ethyl acetate, ethanol, and hexane fractions, as shown in Figure 1. The highest TFC was found in the root-based ethyl acetate fraction (103.76 ± 14.8 mg RE g^−1^) and the aerial ethanol fraction (97.61 ± 10.65 mg RE g^−1^). Similarly, the highest TFC was found in the ethyl acetate fraction of *C. gigantea* ash (184.28 ± 11.64 mg RE g^−1^), followed by the ethyl acetate fractions of *C. gigantea* (112.48 ± 4.28 mg RE g^−1^) and *C. procera* (118.89 ± 0.44 mg RE g^−1^).

### 2.5. Detection of Total Polyphenolic Content (TPC) of Plants

**Figure 2:**
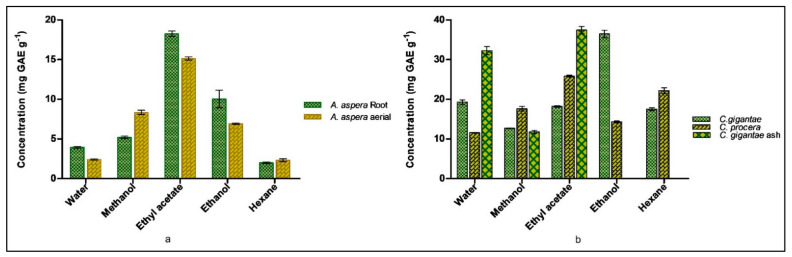
Total Flavonoid content determination (**a**) *A. aspera* (**b**) *C. gigantea* and *C. procera*.

Polyphenolic compounds are key constituents, known to protect plants from reactive oxygen species. This feature enables the extracts to act as reducing agents and as free radical scavengers [39,40]. The total phenolic content (TPC) of *A. aspera* (aerial and root), *C. procera*, *C. gigantea* and *C. gigantea* ash parts were analyzed in water, methanol, ethyl acetate, ethanol, and hexane fractions, shown in Figure 2. The highest TPC was found in the ethyl acetate fraction of *A. aspera* i.e., 15.1 ± 0.36 mg GAE g^−1^ and 18.27 ± 0.56 mg GAE g^−1^ in the aerial and root parts, respectively. *C. gigantea*, *C. gigantea* ash and *C. procera* showed the highest TPC measures (in mg GAE g^−1^) in the ethanol fraction (36.47 ± 0.93), the ethyl acetate fraction (37.45 ± 0.94), and the ethyl acetate fraction (25.85 ± 0.22), respectively.

### 2.6. Minimum Inhibitory Concentrations (MICs)

Extract codes 3 and 23 of the ethyl acetate fractions of *A. aspera* aerial and *C. gigantea* flower ash were found to be active against the *M. tuberculosis* H_37_Rv ATCC 27294 strains, respectively, having an MIC value of 64 mg/L. The rest of the extracts were inactive against tuberculosis and non-tuberculosis strains and showed the MIC value > 64 mg/L. None of the compounds screened were active against non-tuberculous *Mycobacterium*. The known anti-tuberculosis drugs for virulent strains, such as isoniazid, rifampicin, streptomycin, ethambutol showed significant MIC against *M. tuberculosis* H_37_Rv, and levofloxacin (drug used for avirulent strains) showed significant MIC values against all the tested avirulent strains [41,42] (Table S1).

### 2.7. Gas Chromatography-Mass Spectrometry (GC-MS) Studies

**Figure 3:**
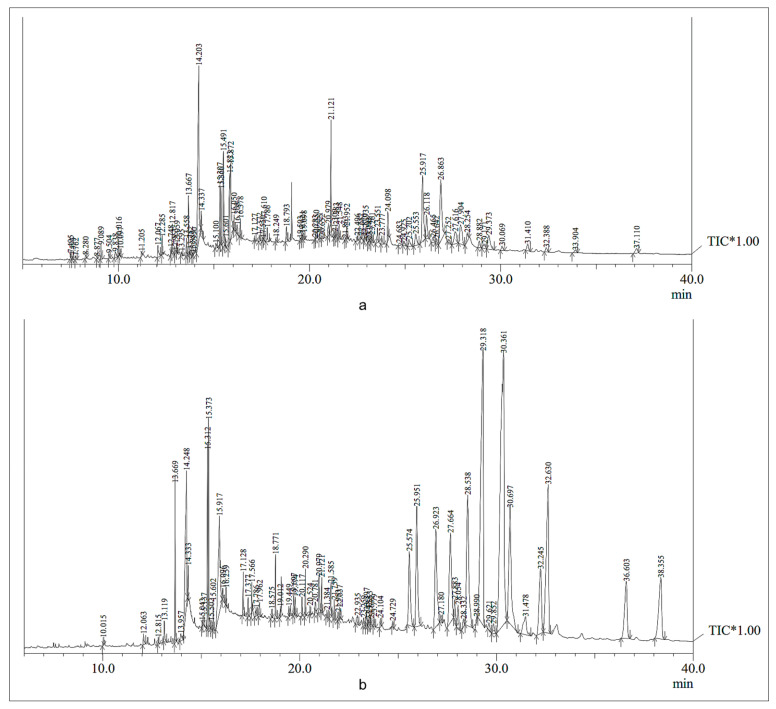
GC-MS chromatogram of ethyl acetate fractions of (**a**) *A. aspera* (aerial) and (**b**) *C. gigantea* (flower ash). TIC* indicates Total Ion Chromatogram.

To get the chemical profile of the phytoconstituents, GC-MS analysis of the ethyl acetate plant extracts was performed, due to these having the occurrence of highest TPC and TFC contents. The chromatogram obtained from GC-MS analysis of the ethyl acetate fractions of *A. aspera* and *C. gigantea* plants showed 87 and 68 peaks, respectively. The height of the individual peak resembled the comparative concentration of the compound in the extract. The GC-MS analysis chromatogram is shown in Figure 3. The studied chromatogram peak identified the phytochemical constituents from the ethyl acetate fractions of the aerial part of *A. aspera* (Table S3) and the flower ash of *C. gigantea* (Table S4).

### 2.8. Assessment of Multitarget Signature Mtb Proteins: An In-Silico Approach

Mycobrowser was analyzed for the availability of genes or proteins responsible for mycobacterial virulence and these were categorized into virulence, detoxification, and adaptation categories [43]. The Venn-diagram used to categorize V.D.A category and known *Mtb* PDB structure proteins is provided in the supporting material (Figure S1). The PDB structures were assessed by employing different in silico methods (Table 3). The crystal structures of the selected *M. tuberculosis* H_37_Rv proteins (PDB: 7LD8, 4Q4N, 5XE2, 4LJ1, 1TQ8, 4CHG, 5WZ4, 2JAX, 5SV2, 6L2A) [44] are provided in Figure S2.

#### 2.8.1. Physiochemical Parameters

**Figure 4:**
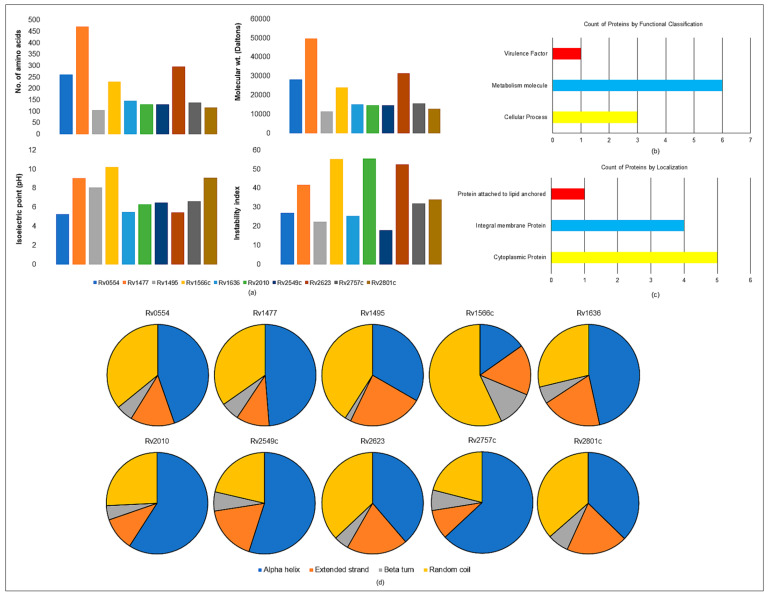
The selected virulence, detoxification, adaptation (VDA) category of *M. tuberculosis* H_37_Rv proteins. (**a**) Physiochemical parameters of VDA proteins (**b**) Functional classification of VDA proteins (**c**) Subcellular localization of VDA proteins and (**d**) Secondary structure analysis of VDA proteins.

Mycobacterial virulent proteins are the major factors that aid in pathogenesis. The amino acid sequence of virulent proteins was obtained from the Mycobrowser database [55]. The Protparam tool was used to calculate the physicochemical parameters for these proteins, as shown in Table S4 [56]. The physiochemical parameter of the proteins calculated as the isoelectric points (pIs) of *Rv1477*, *Rv1495*, *Rv1566* and *Rv2801c* were greater than 7 (pI > 7), which means these proteins had more basic amino acids. The instability index value was also calculated and four unstable proteins (Instability index > 40) were found, viz. *Rv1477*, *Rv1566c*, *Rv2010*, and *Rv2623*. The calculated aliphatic index of some of these proteins were very high, which means they were thermostable. The GRAVY (grand average hydropathy) value was also determined, which indicated that *Rv0554*, *Rv1636*, *Rv2010*, and *Rv2623* were polar, and rest of the six proteins were non-polar (Figure 4a).

#### 2.8.2. Functional Classification

The protein targets were classified functionally under three categories, viz. virulence factors, proteins involved in metabolism, and those involved in cellular processes, and were regarded as an effective target for emerging TB treatments [43]. VICMpred webserver predicted six proteins involved in metabolism, three in cellular processes, and one protein as a virulence factor (*Rv0554*) [57,58,59,60]. The comprehensive analysis of these functional proteins is shown in (Figure 4b).

#### 2.8.3. Subcellular Localization

For forecasting a protein’s function, it is crucial to know where it is located. We used the TBpred server in this investigation to predict localization, which is based on the support vector machine (SVM)-based subcellular localization prediction of the mycobacterial protein [61,62]. The protein’s location in the cytoplasm, integral membrane, lipid anchoring, and secretory pathways is predicted. The cytoplasmic proteins (Rv1636, Rv2010, Rv2549c, Rv2623, Rv2757c), integral membrane proteins (Rv0554, Rv1477, Rv1566, Rv2801c), and lipid anchored proteins (Rv1495) are displayed in (Figure 4c). The prediction accuracy was 86.62%.

#### 2.8.4. Secondary Structure Prediction

The two-dimensional (2D) structure prediction was performed by the SOPMA webserver for all the ten proteins. The SOPMA server is a straightforward and accurate method for predicting the 2D structure of a protein. The predicted 2D structures with default parameters were set to analyze the structural patterns of the proteins. The structural studies concluded that these proteins have more alpha helix regions compared to extended strands in beta-sheet. The details of these predicted 2D structures are given in Table S6 and the schematic representation of the 2D representation is in Figure 4d.

#### 2.8.5. Phylogenetic Analysis

**Figure 5:**
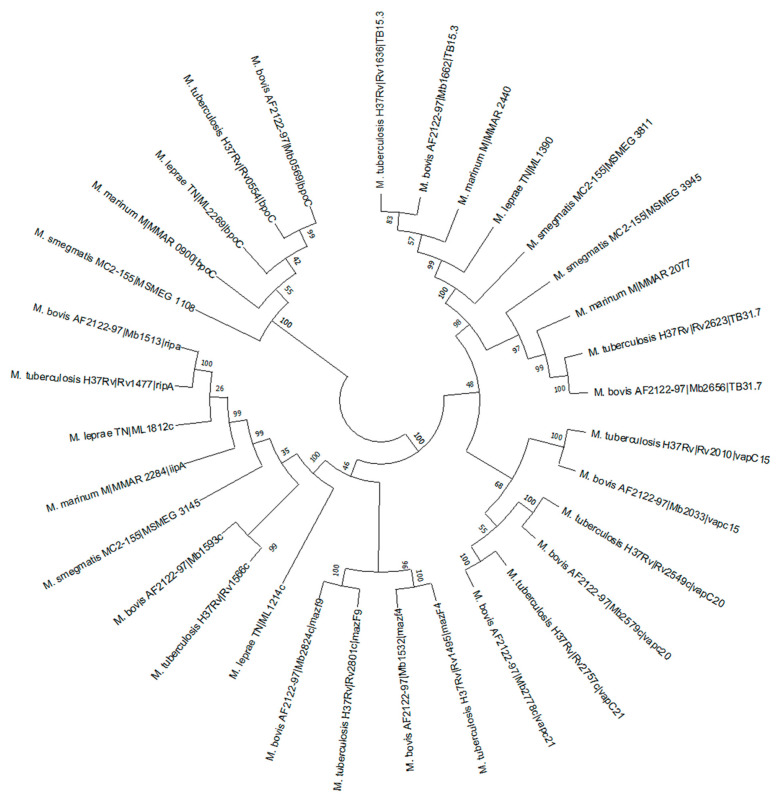
Phylogenetic tree depicting the relationships between virulence categories proteins in *M. tuberculosis* H_37_Rv.

Phylogenetic analysis of all ten selected genes was conducted using the Mega11 program. The homologs in different mycobacterial species were analyzed and the pathlengths were specified for each interaction, and are shown in Figure 5.

#### 2.8.6. PPI Network Analysis

**Figure 6:**
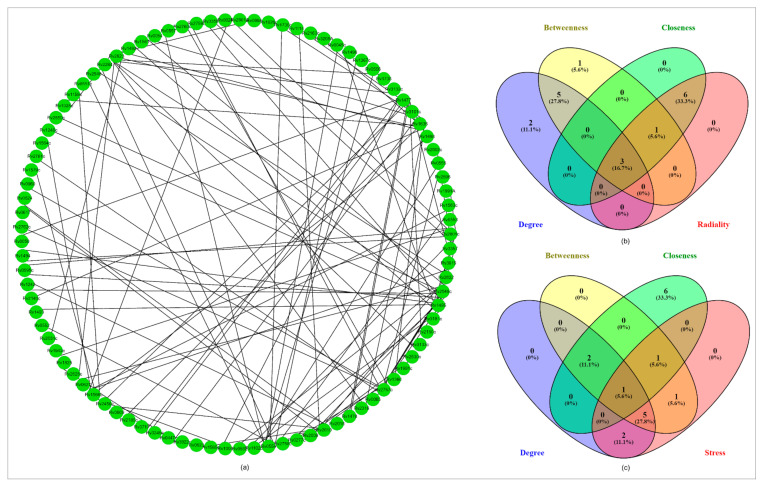
Network analysis: (**a**) PPI network of the selected ten virulent genes which were involved in significant pathways comprised a total number of 93 nodes and 101 edges. (**b**) Venn plot showing the intersection of degree-based radiality category found 3 genes. (**c**) Venn plot showing the intersection degree-based stress category found 3 genes.

The protein-protein interaction (PPI) network analysis was done using the STRING v11.0 database. The input proteins showed a confidence score of 0.6–0.9. In the first category of “betweenness, closeness, radiality and degree”, there were 3 genes found in the intersection (Rv1636, Rv2623 and Rv1566). In the second category of “betweenness, closeness, stress and degree” only one gene (Rv1636) was found in the intersection, as shown in Figure 6.

#### 2.8.7. Structural Classification of the Selected VDA Proteins

*Rv0554* is an integral part of the menaquinone regulatory operon in Mycobacterium. Menaquinone is a crucial factor in the mycobacterial electron transport chain [45]. *Rv1477* is a mycobacterial resuscitation promoting factor interacting protein (*RipA*) and participates in the cleavage of peptide cross linkages between peptidoglycans similar to other cleavage enzymes, such as *OwlT* and *Spr* in *B. subtilis* and *E. coli*, respectively [46]. *Rv1495* is designated as *mazEF*, which is a subcomponent of the type II toxin–antitoxin system in mycobacteria [47]. *Rv1566c* (*RipD*) is a predicted peptidoglycan specific peptidase of the NlpC/p60 family. The unusual peptide linkages of *Mycobacterium* i.e., L-D and D-D and other iso-peptide linkages makes the peptide resilient to cleavage by mycobacterial peptidases [48]. *Rv1636* is a universal stress protein (USP) in *M. tuberculosis*. In *M. tuberculosis*, it is classified into class I USP, based on the presence of only a single conserved domain of USP, which is of similar size to *UspA*. CAMP is bound to *Rv1636*, which regulates the signaling associated with the cAMP molecule [49]. *Rv2010* of *M. tuberculosis* codes for *VapC*-15 toxin. These proteins belong to the PIN domain family proteins and contain ribonuclease activity [50]. *Rv2549c* is designated as *VapC20* of *M. tuberculosis* and along *VapB*, it forms *VapBC* toxin antitoxin complex [51]. *Rv2623* is a universal stress protein of *M. tuberculosis*, that helps mycobacteria in different stress conditions and is also important for mycobacterial growth and persistence [52]. *Rv2757c* of *M. tuberculosis* codes for *VapC*-21 toxin. These proteins also belong to the PIN domain family proteins and contain ribonuclease activity [53]. *Rv2801c* encodes for the *MazF*-mt1 protein of *M. tuberculosis*. *MazF* is a known component of the *MazEF* toxin–antitoxin system in many prokaryotic cells [54].

#### 2.8.8. Validation of the Selected Proteins’ Structures

The PROCHECK server was used to validate the models, and a Ramachandran plot of the projected models revealed that the modeled structure of mycobacterial virulence proteins had ~90% residues in the most favored region, indicating the modeled structure was good [63,64] (Table 4).

#### 2.8.9. Molecular Docking

**Figure 7:**
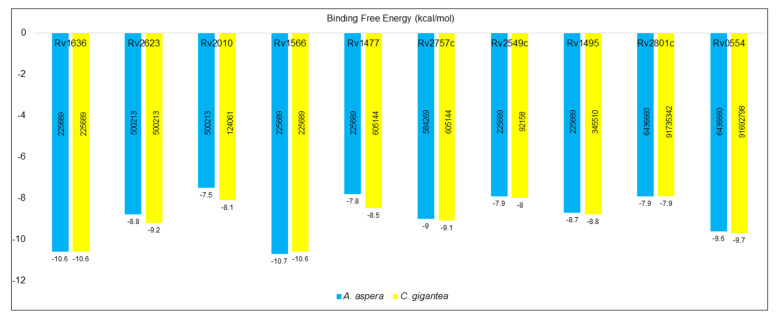
Comparative docking score analysis of the VDA proteins against top hit phytoconstituents.

**Figure 8:**
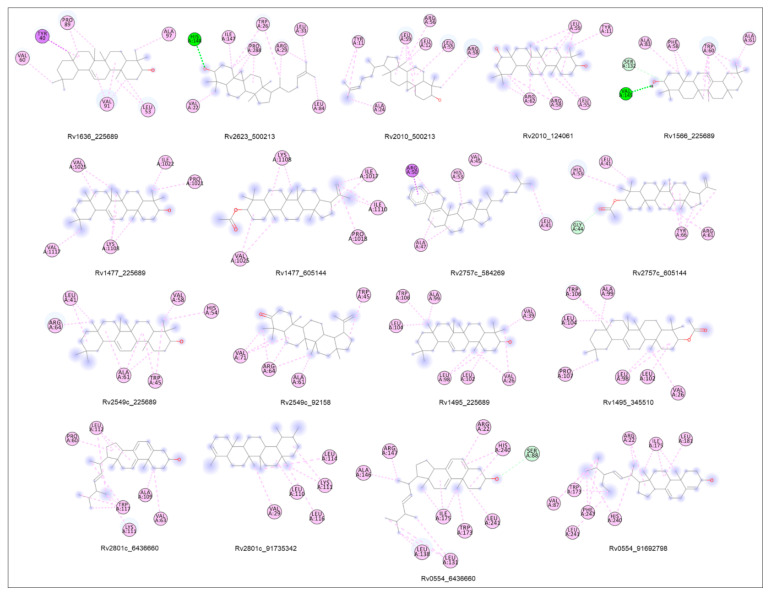
Docking interaction analysis: 2D structural representation of Protein-ligand complexes having pi-Alkyl bonds (purple) and Hydrogen bond (green) along with interacted residues.

The molecular docking analysis was performed by using InstaDock v1.0 software, New Delhi, India (https://hassanlab.org, accessed on 1 April 2022), ChemBioDraw Ultra 14.0, in-house python script and Discovery Studio as mentioned in the experimental section [65,66,67,68,69]. It included the fundamental orientations between the receptor and the ligands (plant extract phytoconstituents). The 10 shortlisted proteins were used for molecular docking in order to explain how these proteins interact with the phytoconstituents identified in the ethyl acetate aerial part of *A. aspera* and flower ash extract of *C. gigantea*. The high negative docking score (binding free energy, kcal/mol) indicated that their binding was steady. In Table S7, the comparative analysis of docking results on the multitarget proteins is provided. The docking scores are shown in Figure 7. The 2D structure of the phytoconstituents is provided in Figure S3. The selected top hit phytoconstituents had binding free energies ranging from −7.5 to −10.6 kcal/mol, and the correspomding proteins were chosen for further studies. *β*-amyrin (PubChem CID: 225689) was found to have higher binding free energies against *Rv1636*, *Rv1566*, *Rv2549c*, and *Rv1495* proteins. The 3D crystal structures of VDA proteins, showing different conformational changes, were identified and structural analysis was carried out using a PyMOL visualizer (Figure 8).

#### 2.8.10. Determination of the Selected Phytoconstituents’ Drug Abilities

The drug-likeness properties of the selected phytoconstituents were assessed using the Swiss ADME, pkCSM and PASS webservers [70,71,72]. The top 10 hit compounds’ physicochemical or drug likeness properties demonstrated that most of the phytoconstituents followed Lipinski’s rule. All the selected compounds’ physiochemical parameters are shown in Table 5. The ADMET properties of the identified phytoconstituents were further investigated in order to rule out any potentially harmful patterns in the molecular structures (Table 6).

A prediction of structure activity relations (SARs) was performed, by a machine learning program using the PASS online webserver, to investigate the biological activities of the selected phytoconstituents. *β*-amyrin was shown to have multiple biological activities, such as insulin promoter, caspase-3 stimulant, transcription factor NF kappa B stimulant, muco-membranous protector, hepatoprotection, apoptosis agonist, antineoplastic, oxidoreductase inhibitor, membrane integrity antagonist, and chemoprevention, with a Pa score ranging from 0.903 to 0.977. The biological activity predictions of all the ten phytoconstituents are provided in Table S8.

Moreover, *β*-amyrin, in combination with lupeol, was reported to have antibacterial activity, including antimycobacterial activity. The mixture of both compounds showed modest antibacterial activity against most of the bacteria, with MIC of 62.5 µg/mL, for *Staphylococcus aureus*, *Pseudomonas aeruginosa*, *Mycobacterium fortuitum* and *Mycobacterium smegmatis*. *β*-amyrin is abundantly found in plants with varied pharmacological activities. The compounds revealed after GC-MS analysis were used for molecular docking analysis with 10 shortlisted proteins. The docked complexes showed that most of the proteins had significant binding affinity with *β*-amyrin. Thus, *β*-amyrin was selected for further MD simulation study.

### 2.9. MD Simulation

The structural conformational stability of a protein-ligand was investigated using molecular dynamics (MD) simulation. The structural insight of Rv1636 and Rv1636 with *β*-amyrin in water at 298 K was analyzed. RMSD, RMSF, Rg, SASA, and funnel energy of landscape (FEL) were tracked throughout 50 ns [73,74,75,76].

#### 2.9.1. Average Potential Energy of System

The average potential energy of Rv1636 and Rv1636_*β*-amyrin complex were monitored to ensure that the system was equilibrated. A constant fluctuation for each system at constant temperature (298 K) and pH (7.0), indicated a steady and accurate MD simulation. The average potential energy of *Rv1636* was −0.061205 × 10^7^ kJ/mol and for the Rv1636_*β*-amyrin complex it was −1.48416 × 10^7^ kJ/mol.

#### 2.9.2. Root Mean Square Distance (RMSD)

**Figure 9:**
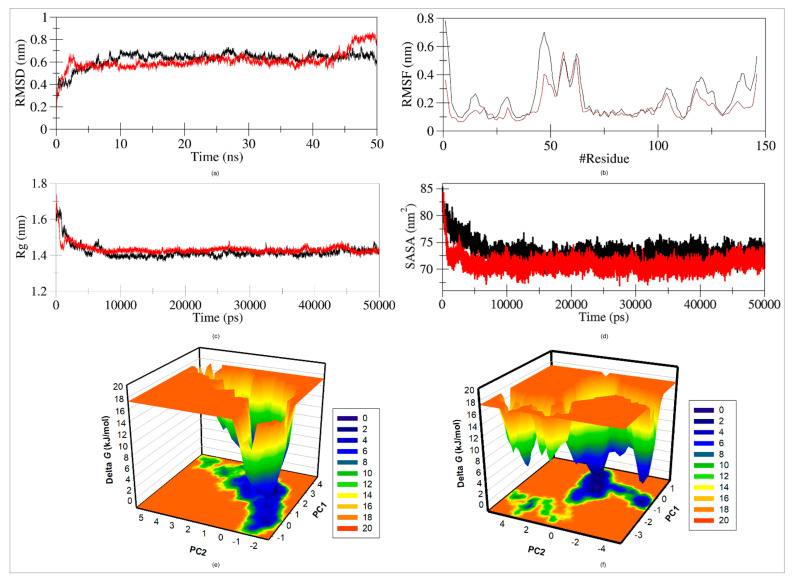
Structural dynamics of universal stress protein (Rv1636) upon *β*-amyrin binding as a function of time. (**a**) RMSD plot of Rv1636 in complex with *β*-amyrin. (**b**) RMSF plot of Rv1636 in complex with *β*-amyrin. (**c**) Structural compactness and folding Rv1636 upon *β*-amyrin. (**d**) SASA plot of Rv1636 as a function of time before and after *β*-amyrin binding. (**e**) The time evolution of projections of trajectories on both EVs. The free energy landscapes of free Rv1636 protein (**f**) The free energy landscapes of free Rv1636 with *β*-amyrin.

The interaction of a ligand with a protein can result in considerable conformational change in the structure. The RMSD was measured as a function of time with respect to the initial conformation and is illustrated in Figure 9a. The RMSD average value for *Rv1636* was 0.71379 nm and for the Rv1636_*β*-amyrin complex it was 0.61481 nm. The RMSD plot evidently implied that the Rv1636 and Rv1636_*β*-amyrin complexes were stable during the simulation time frame till 45 ns, but after 45 ns there was some hindrance showing fluctuation in the Rv1636_*β*-amyrin complex, as compared to Rv1636.

#### 2.9.3. Root Mean Square Fluctuation (RMSF)

The RMSF of the Rv1636 and Rv1636_*β*-amyrin complexes were displayed as a function of residue number to obtain the average fluctuation of all residues throughout the simulation time (Figure 9b). The RMSF revealed that residual fluctuations existed in various regions. These residual fluctuations were observed to be reduced when Rv1636 and *β*-amyrin was bound, rather than when the Rv1636 protein was alone.

#### 2.9.4. Radius of Gyration

Rg is the RMS distance between a group of atoms and their collective center of mass, and it is linked to a protein’s tertiary structure stability. It is one of the most extensively used criteria for determining the compactness of a protein structure. The values of Rg of Rv1636 and the Rv1636_*β*-amyrin complex were 1.42461 nm and 1.43199 nm, respectively. A minor increase in the Rg values of the Rv1636_*β*-amyrin complex was directly in agreement with the RMSF values. The Rg plot suggested that Rv1636 protein was stably folded with the R1636_*β*-amyrin complex, as shown in Figure 9c.

#### 2.9.5. Solvent Accessible Surface Area (SASA)

Solvent accessible surface area (SASA) analysis was performed to investigate protein folding behavior and stability. While studying the plot, no significant changes in SASA values were observed during the simulation time, suggesting a stable complex. The SASA of native protein was found to be 72.4474 nm^2^ and the SASA of the protein-*β*-amyrin complex was 70.9425 nm^2^. A slight decrease in the average SASA signified enhanced packing of the protein-ligand complex, which could also be correlated with the Rg (Figure 9d).

#### 2.9.6. Free Energy Landscape (FEL)

The free energy landscape mechanism was used to define the protein folding into native conformation and protein denaturing landscapes. The two PCs were used to design the FELs and energy minima of Rv1636 and the Rv1636_*β*-amyrin complex. C-alpha atoms were focused to project the conformational sampling of Rv1636 and the Rv1636_*β*-amyrin complex, as illustrated in Figure 9e,f. The FEL plot showed that the binding of *β*-amyrin to Rv1636 slightly distorted the complex size and position of the phase’s minimum. A deep blue plot in Rv1636 free landscape signified the conformation with less energy towards native conformation. The plot showed that Rv1636 showed single global minima confines with single basin. On the other hand, the Rv1636_*β*-amyrin complex also showed single global minima but with more than 1 basin. Finally, this FEL study concluded that the binding of *β*-amyrin to Rv1636 did not cause protein unfolding throughout the simulation run.

## 3. Discussion

The medications used for TB were beneficial in the past but, at present, the insufficiency of these medications has raised the need for the identification and discovery of novel therapeutics. *A. aspera* is a widely known medicinal plant with various anti-bacterial activities. The plant is famous for its contents of alkaloids, saponins, carbohydrates, glycosides, flavonoids, tannins, and triterpenoids [11,12]. *C. gigantea* is also a traditional medicinal herb, used to cure common ailments, such as fevers, cough, cold, asthma etc. [20]. The root bark is an expectorant, febrifuge, anthelmintic, depurative, and laxative. Asthma, bronchitis, and dyspepsia are treated with the powdered root. Paralysis, arthralgia, swellings, and intermittent fevers can all be treated with the leaves. The flowers have a range of properties, being bitter, digestive, astringent, stomachic, anthelmintic, and tonic [22] and make a well-known homoeopathic remedy [22]. *C. procera* is a shrub with purgative, anthelmintic, anticoagulant, palliative (for respiratory and blood pressure disorders), antipyretic, analgesic and neuromuscular blocking properties [24]. The family members of this plant are high in cardiac glycosides [25,26].

The aerial and root parts of *A. aspera* and the flowers of *C. gigantea* and *C. procera* are widely grown, and are commonly used herbal medications [33]. The plants are distinguished through the different appearances of their flowers [34]. The plant parts were used to prepare the extracts in hexane, ethyl acetate, ethanol, methanol and water. The phytochemical analysis showed the presence of flavonoids, alkaloids, phenol, anthraquinone, terpenoids, tannins, steroids, saponins, carbohydrates, glycosides in the extracts [35]. Many flavonoids are key components of medicinal plants and are employed in the regulation of inflammation and cancer prevention, due to their ubiquity in the human diet [40]. The highest total flavonoid content in *A. aspera* was found in the root ethyl acetate fraction (103.76 ± 14.8 mg RE g^−1^) and the aerial ethanol fraction (97.61 ± 10.65 mg RE g^−1^). The highest TFC was found in the ethyl acetate fraction of *C. gigantea* ash (184.28 ± 11.64 mg RE g^−1^) (Figure 1). The total polyphenolic content of the plant aerial and root parts was analyzed in water, methanol, ethyl acetate, ethanol and hexane fractions. Polyphenols are important components of plants as they defend plants from reactive oxygen species [39]. The highest TPC was found in the ethyl acetate fraction i.e., 15.1 ± 0.36 mgGAE g^−1^ and 18.27 ± 0.56 mgGAE g^−1^ in the aerial and root parts of *A. aspera*, respectively (Figure 2). The flavonoid and phenolic content of the extracts was higher in the ash of *C. gigantea*, indicating heating the extracts caused solubilization of these organic compounds. *M. tuberculosis* H_37_Rv ATCC 27294, *M. fortuitum* ATCC 6841, *M. abscessus* ATCC 19977 and *M. chelonae* ATCC 35752 were used to determine the minimum inhibitory concentrations of the extracts against the mycobacterial strains. *A. aspera*’s aerial ethyl acetate fraction (3) and *C. gigantea*’s flower ash ethyl acetate fraction (23) was found to be active against the *M. tuberculosis* H_37_Rv ATCC 27294 strain with an MIC value of 64 mg/L. The non-tuberculous bacteria showed the maximum growth against all extracted compounds (Table S1). Anti-tuberculosis drugs, such as isoniazid, rifampicin, streptomycin, and ethambutol, with MIC values of 0.03 mg/L, 0.3 mg/L, 1 mg/L, and 1 mg/L, respectively, were used as positive control [41,42].

As the ethyl acetate fraction of *A. aspera* and *C. gigantea* plants were the most effective fractions in containing the flavonoid, phenolic content and bacteriostatic effects against *M. tuberculosis* H_37_Rv, it was further analyzed by GC-MS for the detection of various phytoconstituents (Figure 3). The presence of the compounds, as confirmed in the GC-MS analysis, are listed in Tables S3 and S4.

The mycobacterial proteins were categorized into virulence, detoxification, adaptation (VDA) categories, and the proteins having known structural information. The VDA category is a vast family of proteins that participates in maintaining mycobacterial metabolism and, therefore, targeting these proteins would be an asset in targeting mycobacterial cells [43]. After the evaluation of the complete mycobacterial database, 238 proteins were found to belong to the VDA category and 135 proteins had available structural information in the RSCB-PDB databank. In Table 3, a counteractive study on these proteins led to the determination of 10 proteins, which were *Rv1477*, *Rv1495*, *Rv1566*, *Rv2801c*, *Rv2010*, *Rv2623, Rv0554*, *Rv1636*, *Rv2549c*, and *Rv2757c* [44]. *Rv0554* is an integral part of the menaquinone regulatory operon in Mycobacterium. Menaquinone is a crucial factor in the mycobacterial electron transport chain. There are many genes that were identified, which participate in the menaquinone biosynthesis pathway and the operon is present between *Rv0534c* and *Rv0558*. A gene known as *yfbB* in *E. coli* is known as *menH* and *Rv0554* in *M. tuberculosis* and was predicted to encode an enzyme with a similar function to *menH*. Structurally, it was categorized in the alpha beta hydrolase fold of *E. coli* and is the only protein which is located nearest to the *M. tuberculosis* menaquinone synthesis pathway [45]. *Rv1477* is a mycobacterial resuscitation promoting factor interacting protein (*RipA*) that participates in the cleavage of peptide cross linkages between peptidoglycans similar to other cleavage enzymes, such as *OwlT* and *Spr* in *B. subtilis* and *E. coli*, respectively. *Rv1477* was found to colocalize at bacterial septa with resuscitation promoting factor B (*RpfB*). This protein is important for mycobacterial growth as the depletion strains of this protein in *M. smegmatis* showed abnormal phenotype and decreased growth pattern and, therefore, this protein is a wonderful candidate for drug target strategies [46]. *Rv1495* is designated as *mazEF*, which is a subcomponent of type II toxin antitoxin system in mycobacteria. Out of 7 homologs of *mazF* which are identified in *M. tuberculosis*, four homologs comprise endoribonuclease activity. *MazF* acts as a toxin and it recognizes and cleaves to the intracellular RNA sequence in a ribosome-independent manner. As it is a sequence specific ribonuclease, it inhibits translation with a lesser degree than other non-specific toxins. Previous studies have also reported extracellular death factor functioning in mediated quorum sensing [47]. *Rv1566c* (*RipD*) is a predicted peptidoglycan specific peptidase of NlpC/p60 family. The unusual peptide linkages of *Mycobacterium* i.e., L-D and D-D and other iso-peptide linkages makes the peptide resilient to cleavage by mycobacterial peptidases. However, during the cell division process, the mycobacterial cells produce specific peptidases that weaken these linkages and help in generation of daughter cells. *Rv1566c* is such a specific peptidase. The known specific peptidases in mycobacterium are *RipA* and *RipB*, which cleave the peptide stem between D-glutamic acid and meso-Dap residue. *Rv1566c* containing peptidoglycan specific peptidase domain has 52% and 51% similarity with the *RipB* and *RipA* proteins, respectively. The *Rv1566c* is the first example of a peptidase domain, which binds to the peptidoglycan in a non-catalytic manner, and this feature is specific to mycobacteria only [48]. *Rv1636* is a universal stress protein (USP) in *M. tuberculosis*. In *M. tuberculosis* it is classified into class I USP, based on the presence of only a single conserved domain of USP which is of similar size of *UspA*. Rv1636 amino acid sequence contains the GXXG-9X-G-S/T conserved domain. The exact role of *Rv1636* in stress condition is yet to be detected but it was predicted that this protein might exclusively be expressed in hypoxia and other stress conditions. One interesting feature of *Rv1636* is its cAMP binding activity. The significant fraction of intracellular cAMP bound to *Rv1636* regulates the signaling associated with the cAMP molecule [49]. *Rv2010* of *M. tuberculosis* codes for *VapC*-15 toxin. These proteins belong to the PIN domain family proteins and contain ribonuclease activity. *VapC* toxin is deleterious to cells, but its effect gets neutralized by *VapB* antitoxin. This protein works in a similar manner to T4 RNase and Mja FEN-1 endonuclease. *VapBC* complex is a significant toxin–antitoxin system and an important participator in mycobacterial metabolism [50]. *Rv2549c* is designated as *VapC20* of *M. tuberculosis* and along *VapB* it forms the *VapBC* toxin–antitoxin complex. *VapC20* interacts with its cognate partner *VapB20* to form a stable complex. Both proteins in their individual states are present in dimer states, which form stable homo-tetramers or homo-octamers upon interaction. *Rv2623* is a universal stress protein of *M. tuberculosis* that helps mycobacteria in different stress conditions. This protein is also important for mycobacterial growth and persistence. *Rv2623* protein is a highly induced protein of mycobacteria in response to stress conditions, such as hypoxia and nitrosative stress, which the bacteria face in infected host cells. Apart from its role as a USP, this protein also has the ability to bind ATP. *Rv2623* also interacts with *Rv1747*, which is an ABC transporter protein and helps in exporting lipo-oligosaccharides to negatively regulate mycobacterial growth [52]. *Rv2757c* of *M. tuberculosis* codes for *VapC*-21 toxin. These proteins belong to the PIN domain family of proteins and have ribonuclease activity. *VapC* toxin is deleterious to cells, but its effects become neutralized by *VapB* antitoxin. *VapC21* is similar in function to the other known *VapC* proteins of *M. tuberculosis* [53]. *Rv2801c* encodes for *MazF*-mt1 protein of *M. tuberculosis*. *MazF* is a known component of *MazEF* toxin–antitoxin system in many prokaryotic cells. *MazEF* is part of the TA system that forms persister cells of *M. tuberculosis*. *M. tuberculosis* has ten such *MazEF* proteins from numbers 1 to 10 and all *MazF* proteins are RNases. *MazF*-mt1 specifically cleaves mRNA. *MazF* family members play important roles in antibiotic and immune tolerance mechanisms [54].

In silico characterization was performed to determine the secondary structure, polarity, instability index and localization of all the selected 10 proteins. *Rv1477*, *Rv1566c*, *Rv2010*, and *Rv2623* were found to be unstable proteins, based on their instability indices, which were based on protein sequence information. *Rv2010* and *Rv2623* were categorized into polar proteins and, therefore, these proteins might be more vulnerable towards surrounding nature (Figure 4a) [56]. Rv0554 was also found to be a non-essential gene for mycobacterial growth, but it is listed as an important virulence factor that codes for a peroxidase. Most of the proteins participated in mycobacterial metabolism (Figure 4b) [57]. Rv1495 was found to be a lipid anchored protein, and it is a probable toxin (MazF4). Rv1636, Rv2010, Rv2549c, Rv2623, Rv2757c are cytoplasmic proteins and, therefore, components of the secretory system of mycobacteria (Figure 4c) [58]. The secondary structure analysis showed that most of the regions of the proteins were comprised of an alpha helical pattern, which confirmed the stable structural state of the proteins (Figure 4d) [59].

The phylogenetic analysis was performed by Mega11 program and showed close proximity of Rv1636 of *M. tuberculosis* H_37_Rv and *Mycobacterium bovis* Rv1662, where both code for USP TB15.3. *M. tuberculosis* H_37_Rv Rv1636, *M. marinum* 2440, *Mycobacterium bovis* 1662, *M. leprae* 1390, *M. smegmatis* mc^2^155 3811 showed significant identity percentages among their USP domain (Figure 5) [60]. The protein interaction was determined by STRING server and properties like betweenness, closeness, radiality, stress and degree were used as the parameters for the interactive analysis. The analysis configured three proteins Rv1636, Rv2623 and Rv1566 in betweenness, closeness, radiality, and degree category, whereas Rv1636 was the only protein which was highlighted in the stress parameter (Figure 6) [61,62].

Molecular docking studies were executed to determine the highly interactive compound for their binding capacity with the proteins. The structure of the compound is mentioned in (Figure S3). *β*-amyrin (PubChem CID: 225689) was found to have higher binding free energies against *Rv1636*, *Rv1566*, *Rv2549c*, and *Rv1495* proteins (Figure 7). *β*-amyrin strongly integrated with most of the proteins and the interaction involved the pi-alkyl bonds, and hydrogen bonds (Table S7, Figure 8). The ADMET properties confirmed that shortlisted and highly interactive compounds can be a putative drug candidate, as they passed all the qualifying parameters (Table 5 and Table 6) [70,71,72]. Most of the proteins showed high and significant binding affinity with *β*-amyrin, and, thus, it was selected for further analysis [77].

*Rv1636* protein was found to be the top candidate in all the examinations (interaction, docking, biological process etc.), and, therefore, this protein was further analyzed for its stability with *β*-amyrin by molecular dynamic simulation. The RMSD plot showed that the protein and its complex were stable in the initial period till 45 ns, but started to experience a little destabilizing after 45 ns (Figure 9a). This destabilization might be due to the change in the protein structure, as in the instability index *Rv1636* was found to be an unstable protein. The RMSF plot showed instability in most of the residues, whereas the complex showed lesser fluctuation as compared to the protein alone (Figure 9b) [73,74,75]. The SASA result suggested that the binding of *β*-amyrin to the protein stabilizes the complex (Figure 9d) and this stability was further confirmed by Rg plot (Figure 9c) and FEL, which also confirmed the compactness and folding of the protein in complex form with *β*-amyrin (Figure 9e,f) [76].

## 4. Materials and Methods

### 4.1. Plant Collection and Identification

The medicinal plants *Achyranthes aspera*, *Calotropis gigantea* and *Calotropis procera* were collected from a burial ground in Shahjahanpur, Uttar Pradesh, India. The plants were identified by their flowers and inflorescence and the authenticity of these medicinal plants was established from previous literatures [33,34].

### 4.2. Plant Extraction

*A. aspera* aerial and root parts, as well as *C. gigantea* and *C. procera* flowers, were carefully cleaned with running tap water and then with sterile autoclaved water. The material was shade-dried, indelicately pulverised with a motor and pestle, and then extracted. Using a Soxhlet extractor, a weighed amount (500 g) of the substance was extracted using solvents of different polarity, including water, methanol, hexane, ethyl acetate, and ethanol. Nearly 48 extraction cycles were completed, under reduced pressure and at a controlled temperature, using a rotatory evaporator. The extracts were then concentrated, dried, packaged, and kept in a refrigerator at 4 °C for use [35,36].

### 4.3. Secondary Metabolite Identification

To identify various phytoconstituents, all extracts were subjected to a preliminary phytochemical examination using conventional techniques. Many antioxidants, such as alkaloids, terpenoids, saponins, and other compounds with varied pharmacological effects, were found in the plants [37,38].

#### 4.3.1. Alkaloids Presence: Mayer’s Reagent Test

An amount of 5000 µL of extract solution was warmed in a water bath with 2% HCl, and some droplets of Mayer’s reagent was added. The sample was examined for the existence of turbidness or yellow precipitation.

#### 4.3.2. Tannins Presence: Ferric Chloride Test

An amount of 500 µL of plant extract was added to 1000 µL of distilled water and some droplets of ferric chloride were mixed in. The presence of a green black colour showed the presence of tannins.

#### 4.3.3. Saponins Presence: Frothing Test

An amount of 1000 µL plant extract was added to 4000 µL of distilled water and shaken vigorously. The appearance of foam showed the presence of saponins, which persisted for at least 15 min.

#### 4.3.4. Terpenoids Presence: Salkowski Test

Intp 5000 µL extract solution and 2000 µL of chloroform, 3000 µL sulphuric acid was carefully added. The formation of a layer with a greyish colour indicated the presence of terpenoids.

### 4.4. Total Flavonoid Content (TFC)

The TFC of the medicinal plant extracts was determined by the aluminum chloride calorimetric method [39]. A 100 µL solution of 2% aluminum chloride in methanol was added to 100 µL of extract samples. The solution was incubated for 30 min at room temperature (RT) and the optical density was measured at 415 nm. Before adding the aluminum chloride solution, a pre-plate reading was taken. The standard curve was built using five different Rutin concentrations. Extract TFC was measured in mg Rutin equivalents per gram of extract [40].

### 4.5. Total Polyphenolic Content (TPC)

The TPC of the medicinal plant extracts was estimated by slightly changing the Folin-Ciocalteu method used by Siddhuraju et al. [39]. A pre-plate reading was taken and after that 20 µL of each plant extracts were added to 110 µL of ten times diluted newly made Folin-Ciocalteu reagent. After that, 70 µL of sodium carbonate solution was added and incubated for 30 min at RT and the optical density (absorbance) was determined at 765 nm. Gallic acid (GA) was used as a standard to plot standard curves with five different concentrations. The medicinal plant extract TPC was quantified in milligram (mg) of GA equivalents per gram (g) of extract [40].

### 4.6. Minimal Inhibitory Concentration (MIC) Assay

The mycobacterial strains used were *M. tuberculosis* H_37_Rv ATCC 27294, *M. fortuitum* ATCC 6841, *M. abscessus* ATCC 19977 and *M. chelonae* ATCC 35752. The mycobacterial strains were cultured in Middlebrook 7H9 enriched (Difco, Becton, NJ, USA) 10% Oleic acid, Dextrose, 0.2% glycerol, BSA and 0.05% Tween-80 supplemented medium.

The antibacterial susceptibility testing was performed using a broth microdilution technique. Stock solutions of plant extracts and control substances at 10 mg/mL in DMSO were prepared and kept at −20 °C. Bacterial cultures were put into appropriate media and their absorbance was measured at OD_600_, before diluting the culture to reach a concentration of 10^5^ CFU/mL. The plant extracts were evaluated in a two-fold serial dilution method from 64 to 0.5 mg/L, with 2.5 µL of every individual concentration added in each well of Elisa plate. Each well contained bacterial culture around 97.5 µL with the test drug and associated controls. Resazurin-based dye (Thermo Fisher, Waltham, MA, USA) was applied to visually identify active phytoconstituents. The lowest concentration of active substance that prevented observable development after an incubation period was established as the MIC of the active plant extracts. The MIC experiment was done in triplicate and independently on duplicate samples for each drug. The MIC 96 well (Elisa) microtiter plate was incubated for non-tuberculous mycobacteria for 24–48 h and slow growers for 7 days [41,42].

### 4.7. GC-MS Analysis of Plant Extracts

The ethyl acetate fraction of *A. aspera* aerial part and the *C. gigantea* flower part were analyzed using Shimadzu GCMS-QP2010 Ultra, furnished with a Flame Thermionic Detector (FTD detector), to identify the chemical composition of the fractions. Helium gas was employed as the carrier with a 0.7 mL min^−1^ flow rate. The injection temperature was 260 °C, and the preliminary column temperature of 100 °C was kept for two minutes before ramping to 250 °C at a rate of 10 °C min^−1^ and hold on for 19 min before increasing to 290 °C at a rate of 10 °C min^−1^. A solvent delay of 3.5 min was used. Mass spectra were documented in the range of 40–650 m/z and compounds were recognized by using NIST11 Library.

### 4.8. Determination of the Target Proteins: Using In Silico Approaches

To study the mycobacterial target, the proteins of virulence, detoxification, adaptation (VDA) functional category and proteins having PDB structures from the mycobacterial database were selected and an array of in silico analysis was performed. The mycobacterial genome database Mycobrowser was analyzed for the availability of genes responsible for mycobacterial virulence and categorized into virulence, detoxification, adaptation category [43]. *M. tuberculosis* H_37_Rv genome had a total 4173 proteins, out of which 238 proteins belonged to virulence, detoxification, adaptation category. On the other hand, the RSCB-PDB databank contained PDB structures of 135 *M. tuberculosis* H_37_Rv proteins excluding repeated or mutated ones, which were categorized in the mentioned categories (Figure S1). The co-integrative analysis of both these categories showed that there were 10 VDA functional category proteins, having PDB structures that were also present, and these 10 proteins were, therefore, present in both categories. We employed various bioinformatics tools which might empower experimental work to identify the prospective targets of this bacterium and illuminated the efficacy of significant regulators of mycobacterial pathways (Table S2) [44].

#### 4.8.1. Retrieval of the Protein Sequence

The sequence of *M. tuberculosis* H_37_Rv proteins was obtained from the Mycobrowser (Mycobacterial browser) online database, consisting of different types of pathogenic and non-pathogenic mycobacterial strains in a repository for genomic and proteomic comprehensive analysis [55].

#### 4.8.2. Physiochemical Parameters

The proteins identified were investigated for their physiochemical properties. The ProtParam server was used for calculating the theoretical parameters, such as molecular weight, amino acids, pI, instability index etc. [56].

#### 4.8.3. Functional Classification

The primary identifying mechanism for understanding bacterial pathogenesis in prokaryotes is the distinction of virulent and non-virulent proteins. The VICMpred online prediction server was used for functional classification of bacteria using a bi-layer cascade SVM approach, which applies the sequence information for the prediction of different virulent factors. The VICMpred webserver used amino acid sequence in a pattern-based approach that showed extremely important values of functional classification (median values > 1.0) [57].

#### 4.8.4. Subcellular Localization

Protein localization is an important aspect in identifying new drug targets. Since no information regarding the subcellular localization of these protein sequences was available, the TBpred webserver was used to predict the localization of selected proteins. Multiple prediction approaches for analyzing the localization of mycobacterial proteins were used to predict the presence of protein, whether in membrane, cytoplasm, lipid-anchored and secreted categories, based on the scores [58].

#### 4.8.5. Secondary (2D) Structure Prediction

The SOPMA webserver was used to predict the 2D structure of target proteins. This online server is simple and accurate, and predicts different characteristics in secondary structure, such as alpha-helix, beta turns, extended strands or random coil region [59].

#### 4.8.6. Phylogenetic Analysis

Distant and close relatives of virulence proteins were searched in the Mycobrowser database. The boundaries of these genes were specified through the Pfam database and multiple sequence alignments were performed. Phylogenetic analysis was performed by the Mega11 server neighbor joining method [60].

#### 4.8.7. Virulent Genes Regulating Network Analysis

A protein-protein interaction network of virulent proteins was established using STRING v11.5 database, accessed on 7 January 2022 (https://string-db.org/) and visualized by using STRING application, available in Cytoscape. A cutoff score of 0.6–0.9 was selected that showed interaction with high confidence. The studied genes were further analyzed by enrichment analysis by setting a significant statistical threshold less than 0.05. The resulting proteins were then classified by four intrinsic factors, such as “betweenness, closeness, radiality and degree” and betweenness, closeness, stress, and degree. The top virulent proteins were ranked using these factors by the CytoHubba application in the CytoScape [61,62].

#### 4.8.8. Retrieval of the 3D Protein’s Structure

The PDB structure of all the available proteins were retrieved from the RCSB Protein Databank (PDB ID: 7LD8, 4Q4N, 5XE2, 4LJ1, 1TQ8, 4CHG, 5WZ4, 2JAX, 5SV2, 6L2A) [45,46,47,48,49,50,51,52,53,54]. Out of these ten proteins, only *Rv1477* was essential for in vitro growth of *M. tuberculosis* H_37_Rv (PDB ID: 4Q4N). The 3D structure of these proteins was visualized by using PyMOL (Figure S2).

#### 4.8.9. Validation of the Selected Protein’s Structure

The model validation of a protein structure was performed by SAVES6.0 webserver, that estimated various characteristics, especially the stereo-chemical feature of a protein structure by residue geometry. SAVES6.0 server had PROCHECK, and analyzed the Ramachandran plot. It endorsed the protein structure on the premise of φ, ψ values of an individual deposit. The inclusive structure geometry established the validation score of the Ramachandran plot for a protein structure depending on number of amino acids present in favored, allowed, and disallowed regions [63,64].

#### 4.8.10. Molecular Docking

Molecular docking studies were carried out in order to identify the top hit phytoconstituents present in the aerial part extract of *A. aspera* (ethyl acetate fraction) and flower part of *C. gigantea* (ethyl acetate fraction) [65]. The three-dimensional (3D) crystal structure of target proteins (PDB: 7LD8, 4Q4N, 5XE2, 4LJ1, 1TQ8, 4CHG, 5WZ4, 2JAX, 5SV2, 6L2A) was retrieved from RCSB PDB and refined before performing molecular docking. Binding sites/pockets were determined by using CASTp 3.0 server, which examined the geometric and topological properties of the protein structures, including surface pockets, interior cavities and cross channels, as they are fundamentally important for the proteins to carry out their functions. The GC-MS analyzed phytoconstituents (as ligand) were downloaded from PubChem database. The compound structures unavailable in PubChem database were drawn by ChemBioDraw Ultra 14.0 [66]. For the receptor preparation, the water molecules and co-crystallized ligands were removed from the PDB file and polar hydrogens were added. The receptor protein (target) was transformed from pdb format into pdbqt format using in-house protocol [67]. In molecular docking was performed by InstaDock. Results were evaluated from the log files via Python script [68]. The blind docking mechanism was used to explore the binding site(s) in the protein structures. PyMOL was used to visualize protein-ligand interactions. The receptor-ligand complex was prepared by Discovery Studio and 2D interaction of docked conformations was analyzed to understand the ligand binding amino acid residues [69]. The best-fitting conformation related to the binding affinity of the ligand-receptor complex was identified, while keeping the receptor as a rigid entity and ligand as flexible. The top 10 hits showing strong binding affinity to the binding sites were selected.

#### 4.8.11. Determination of the Selected Phytoconstituent Drug-Ability

The physiochemical properties of the chosen compounds were calculated through online SwissADME software. Further, ADME properties and toxicity of the selected compounds were also calculated using the freely available online sever pkCSM. An online server, PASS, was also used for predicting the biological activity of these natural compounds [70,71,72].

### 4.9. MD Simulation

To conduct MD simulations, GROMACS 5.1.2 Bio-Simulation package was used. To clarify the molecular dynamic characteristics and different computations of proteins and ligands employed in this in silico study, the force field GROMOS96 43a2 was used [73]. The receptor-ligand docked complexes files were retrieved using the gmx grep module. To create ligand topology and force-field conditions, PRODRG server was used [74]. To solvate the protein, the water model SPC216 was employed. A 50 ns MD simulation in water at 298 K was used as a control. All protein and ligand atoms were equilibrized in a three-dimensional box with a range of almost 10.5 Å from all side. The protein was thoroughly equilibrated in water, and redundant molecules were removed. To eliminate all poor contacts, energy was minimized for each system with the steepest decline up to a forbearance of 1000 kJ mol^−1^nm^−1^ and the overall charge was neutralized in the system by adding ionic concentrations of NaCl. To perform the simulation, the sizes (x, y, and z) of the simulation frame were established depending on the size and 3D positioning of the protein. All the systems were produced in a specified box, with the protein in the center and water and co-solvents padded around it. The energy minimization method was carried out using the steepest-descent algorithm and conjugate gradient. Two troupe methods, NVT and NPT, were used to equilibrize the system. Before beginning the MD run, environments, such as pH and temperature, were pre-defined. All this evidence was contained in the NVT, NPT, and MD criteria files. The binary trajectory file was generated after the production run for additional examination [75].

## 5. Conclusions

The effect of the phytoconstituents of *C. procera*, *C. gigantea* and *A. aspera* plant extracts on the *M. tuberculosis* H_37_Rv cell proteins was investigated in this study. The phytochemical analysis of all plant extracts showed the presence of a significant content of phenols and flavonoids, especially in the ethyl acetate fraction of *A. aspera* and the ash of *C. gigantea* fractions. The plants extracts were tested against different mycobacterial strains. *A. aspera* aerial and *C. gigantea* flower ash was found to be active against the *M. tuberculosis* H_37_Rv ATCC 27294 strains with an MIC value of 64 mg/L. A multitarget assessment study was used to identify the possible mycobacterial target proteins. Ten proteins, viz. *BpoC*, *RipA*, *MazF4*, *RipD*, *TB15.3*, *VapC15*, *VapC20*, *VapC21*, *TB31.7*, *MazF9*, were found in the intersection of two categories, viz. available PDB dataset proteins and proteins classified in virulence, detoxification, adaptation. In silico characterization identified *TB15.3* (Rv1636) in the intersection of PPI network, which are the universal stress proteins. The phylogenetic analysis showed Rv1636 is a conserved protein among different mycobacterial strains. The molecular docking study of *β*-amyrin revealed its highest binding affinity with *Rv1636*. Furthermore, MD simulation was used to determine the stability and accuracy of the complex and it showed that the complex of *β*-amyrin and *Rv1636* was a stable complex, and the protein did not undergo unfolding during the simulation run. On a final note, this study established a significant bridge in the field of mycobacterial biology, which focused on targeting Rv1636, a universal stress protein of mycobacteria, through natural phytoconstituents.

## Figures and Tables

**Table 1:** Total yield of the extractable components (EC) from *A. aspera* (aerial and root parts) *C. procera* (flower), *C. gigantea* (flower) and *C. gigantea* (flower ash).

S. No.	Extracted Fractions of *A. aspera* Aerial Part (1.5 kg)	EC (in g)	Extracted Fractions *A. aspera* of Roots (300 g)	EC (in g)
1	Methanol	58.85	Methanol	39.93
2	Aqueous	54.60	Aqueous	35.86
3	Hexane	7.14	Hexane	1.82
4	Ethyl acetate	3.18	Ethyl acetate	0.93
5	Ethanol	1.73	Ethanol	0.46
	**Extracted fractions of *C. procera* Flower (200 g)**	**EC (in g)**	**Extracted Fractions *C. gigantea* Flower (200 g)**	**EC (in g)**
1	Methanol	13.26	Methanol	11.33
2	Aqueous	10.83	Aqueous	9.66
3	Hexane	1.02	Hexane	3.82
4	Ethyl acetate	3.56	Ethyl acetate	3.33
5	Ethanol	1.20	Ethanol	1.26
	**Extracted fractions *C. gigantea* Flower (200 g)**	**EC (in g)**		
1	Methanol	12.56		
2	Aqueous	9.89		
3	Ethyl acetate	3.69		

**Table 2:** Phytochemical screening of alkaloids, tannins, saponins, and terpenoids present in the various plant fractions.

S. No.	Phytochemicals	Methanol	Aqueous	Ethanol	EtOAc	Hexane
		Aerial Part Extracts of *Achyranthes aspera*				
1	Alkaloids	+	−	+	−	−
2	Tannins	+	−	−	+	−
3	Saponins	−	−	+	+	−
4	Terpenoids	+	−	+	−	−
		**Roots Extracts of *Achyranthes aspera***				
1	Alkaloids	+	+	−	+	−
2	Tannins	+	−	+	+	−
3	Saponins	+	+	+	−	−
4	Terpenoids	−	+	−	+	−
		**Flower Extracts of *Calotropis procera***				
1	Alkaloids	+	+	+	−	−
2	Tannins	+	+	+	+	+
3	Saponins	+	−	−	−	+
4	Terpenoids	−	+	−	+	+
		**Flower Extracts of *Calotropis gigantea***				
1	Alkaloids	−	+	+	+	−
2	Tannins	+	−	−	−	−
3	Saponins	+	+	−	−	+
4	Terpenoids	+	−	−	+	−

**Table 3:** Assessment of multitarget signature *Mtb* proteins based on virulence, detoxification, and adaptation category.

S. No.	Rv No.	Name	PDB ID	Information	Ref.
1	0554	*BpoC*	7LD8	Possible peroxidase BpoC (Non-essential gene for in vitro growth of H_37_Rv)	[45]
2	1477	*RipA*	4Q4N	Peptidoglycan hydrolase; (essential gene for in vitro growth of H_37_Rv)	[46]
3	1495	*MazF4*	5XE2	Possible toxin MazF4 (non-essential gene for in vitro growth of H_37_Rv)	[47]
4	1566c	*RipD*	4LJ1	Possible *Inv* protein (non-essential gene for in vitro growth of H_37_Rv)	[48]
5	1636	*TB15.3*	1TQ8	Iron-regulated universal stress protein family protein TB15.3 (non-essential gene for in vitro growth of H_37_Rv)	[49]
6	2010	*VapC15*	4CHG	Toxin VapC15 (Non-essential gene for in vitro growth of H_37_Rv)	[50]
7	2549c	*VapC20*	5WZ4	Possible toxin VapC20 (non-essential gene for in vitro growth of H_37_Rv)	[51]
8	2623	*TB31.7*	2JAX	Universal stress protein family protein TB31.7 (non-essential gene for in vitro growth of H_37_Rv)	[52]
9	2757c	*VapC21*	5SV2	Possible toxin VapC21 (non-essential gene for in vitro growth of H_37_Rv)	[53]
10	2801c	*MazF9*	6L2A	Toxin MazF9 (non-essential gene for in vitro growth of H_37_Rv)	[54]

**Table 4:** Ramachandran plot statistics of selected virulent category proteins.

S. No.	Protein	Most Favoured Region	Additional Allowed Region	Generously Allowed Region	Disallowed Region
1	Rv0554	92.1%	7.5%	0.0%	0.4%
2	Rv1477	92.5%	6.9%	0.0%	0.6%
3	Rv1495	95.3%	4.7%	0.0%	0.0%
4	Rv1566c	98.0%	2.0%	0.0%	0.0%
5	Rv1636	91.0%	9.0%	0.0%	0.0%
6	Rv2010	94.2%	5.0%	0.0%	0.7%
7	Rv2549c	95.8%	4.2%	0.0%	0.0%
8	Rv2623	74.5%	22.7%	1.8%	0.9%
9	Rv2757c	96.7%	3.3%	0.0%	0.0%
10	Rv2801c	94.8%	5.2%	0.0%	0.0%

**Table 5:** The physicochemical properties of the selected top hit compounds.

PubChem ID	Name	#M.W.	#Rot. Bond	#HBA	#HBD	LogP
225689	Beta-Amyrin	426	0	1	1	4.74
500213	Handianol	426.72	4	1	1	5.17
584269	-	472.79	5	0	0	5.88
6436660	Dehydroergosterol	394.63	4	1	1	4.68
124061	Olean-12-ene-3, 22-diol	442.72	0	2	2	4.65
605144	-	468.75	3	2	0	4.95
92158	Lupenone	424.70	1	1	0	4.54
345510	Beta-Amyrenyl acetate	468.75	2	2	0	5.19
91537342	24-Norursa-3, 12-diene	394.68	0	0	0	4.76
91692798	Stigmasta-4,7,22-triene-3. alpha.-ol	410.67	5	1	1	4.70

#M.W. (Molecular Weight (Da); #Rot. bond (Rotatable bond); #HBA (hydrogen bond acceptor); #HBD (hydrogen bond donor).

**Table 6:** ADMET properties of the top hit selected compounds.

PubChem ID	Absorption		Distribution (BBB/CNS Permeation)	Metabolism (CYP2D6 Inhibitor)	Excretion OCT2 Substrate	Toxicity A/H/S
	GI abs.	W.S.				
225689	93.733	−6.531	No	No	No	No
500213	95.248	−5.762	No	No	No	No
584269	97.43	−4.664	No	No	No	No
6436660	94.999	−7.112	No	No	No	No
124061	92.522	−6.351	No	No	No	No
605144	98.182	−5.878	No	No	No	No
92158	98.467	−5.828	No	No	No	No
345510	97.342	−6.649	No	No	No	No
91537342	95.778	−6.925	No	No	No	No
91692798	95.604	−6.696	No	No	No	No

GI abs. (Gastrointestinal absorption percentage); W.S. (Water Solubility (log mol/L); BBB/CNS permeation (blood brain barrier/central nervous system); Toxicity A/H/S (toxicity AMES/Hepatotoxicity/Skin sensitization).

## Data Availability

The data that supports the findings of this study are contained within the article and supporting information.

## Supplementary Materials

The following supporting information can be downloaded at: https://www.mdpi.com/article/10.3390/molecules27144581/s1, Table S1: Minimum inhibitory concentration (MIC) of different plants extracts in various solvent fractions against *M. tuberculosis* strains; Figure S1: Venn-diagram to categorize V.D.A category and known *Mtb* PDB structure protein; Figure S2: Crystal structure of selected *M. tuberculosis* H_37_Rv proteins (PDB: 7LD8, 4Q4N, 5XE2, 4LJ1, 1TQ8, 4CHG, 5WZ4, 2JAX, 5SV2, 6L2A); Figure S3: In multitarget protein docking, the top hit selected phytoconstituents; Table S2: List of the bioinformatics tools and databases for learning substantial outcome of Hypothetical protein from *M. tuberculosis* H_37_Rv; Table S3: Phytochemical constituents identified in the ethyl acetate aerial part extract of *A. aspera* using gas chromatography–mass spectrometry; Table S4: Phytochemical constituents identified in the ethyl acetate flower ash extract of *C. gigantea* using gas chromatography–mass spectrometry; Table S5: Physiochemical parameters of selected virulence, detoxification, adaptation category proteins; Table S6: Secondary structure analysis of selected virulent proteins of *Mtb*; Table S7: Selected hits and their binding free energies (kcal/mol) toward multiple target proteins; and Table S8: Selected compounds and their biological properties.
