# Supplementary material for: Potential Efficacy of β-Amyrin Targeting Mycobacterial Universal Stress Protein by In Vitro and In Silico Approach
Source: Molecules. 2022 Jul 18;27(14):4581. doi: 10.3390/molecules27144581 (PMC9320329; doi:10.3390/molecules27144581)
Supplement: Supplementary file 1 [file molecules-27-04581-s001.zip › molecules-1770333-supplementary.pdf]

Supporting information

Potential efficacy of *β*-amyrin targeting mycobacterial universal stress protein by *in vitro* and *in silico* approach

Md Amjad Beg [1], Shivangi [2], Obaid Afzal [3,*], Md Sayeed Akhtar [4], Abdulmalik S. A. Altamimi [3], Afzal Hussain [5], Ali Imam [1], Mohammad Naiyaz Ahmad [6,7], Sidharth Chopra [6,7], Fareeda Athar [1,*]

1. Centre for Interdisciplinary Research in Basic Science, Jamia Millia Islamia, Jamia Nagar, New Delhi 110025, India; md164429@st.jmi.ac.in (M.A.B.); fathar@jmi.ac.in (F.A.); ali.imamuit@gmail.com (A.I.).
2. CSIR-Institute of Genomics and Integrative Biology, Mall Road, Delhi 110007, India; mathuria93shivangi@gmail.com (S.).
3. Department of Pharmaceutical Chemistry, College of Pharmacy, Prince Sattam Bin Abdulaziz University, Al-Kharj 11942, Saudi Arabia; o.akram@psau.edu.sa (O.A.); as.altamimi@psau.edu.sa (A.S.A.A.).
4. Department of Clinical Pharmacy, College of Pharmacy, King Khalid University, Abha 61421, Saudi Arabia; mdhusain@kku.edu.sa (M.S.A.).
5. Department of Pharmaceutics, College of Pharmacy, King Saud University, P.O. Box 2457, Riyadh 11451, Saudi Arabia; afzal.pharma@gmail.com (A.H.).
6. Division of Molecular Microbiology and Immunology, CSIR-Central Drug Research Institute, Sector 10, Janakipuram Extension, Sitapur Road, Lucknow 226031, Uttar Pradesh, India; naiyaz.ahmad@gmail.com (M.N.A.); skchopra007@gmail.com (S.C.).
7. Academy of Scientific and Innovative Research (AcSIR), Ghaziabad 201002, Uttar Pradesh, India; naiyaz.ahmad@gmail.com (M.N.A.); skchopra007@gmail.com (S.C.).

*** Correspondence:** fathar@jmi.ac.in (F.A.); o.akram@psau.edu.sa (O.A.); Tel.: +911126984492 (F.A.); +966115886094 (O.A.)

Table of contents

S. No.	Content	Page No.
1.	**Table S1.** Minimum inhibitory concentration (MIC) of different plants extracts in various solvent fractions against *M. tuberculosis* strains.	S3
2.	**Figure S1:** Venn-diagram to categorized V.D.A category and known *Mtb* PDB structure protein.	S4
3.	**Figure S2:** Crystal structure of selected *M. tuberculosis* $H_{37}Rv$ proteins (PDB: 7LD8, 4Q4N, 5XE2, 4LJ1, 1TQ8, 4CHG, 5WZ4, 2JAX, 5SV2, 6L2A).	S4
4.	**Figure S3**: In multitarget proteins docking, the top hit selected phytoconstituents.	S5
5.	**Table S2:** List of the bioinformatics tools and databases for learning substantial outcome of Hypothetical protein from *M. tuberculosis* $H_{37}Rv$.	S5
6.	**Table S3:** Phytochemical constituents identified in the ethyl acetate aerial part extract of *A. aspera* using gas chromatography-mass spectrometry.	S6-S7
7.	**Table S4:** Phytochemical constituents identified in the ethyl acetate flower ash extract of *C. gigantea* using gas chromatography-mass spectrometry.	S8-S9
8.	**Table S5:** Physiochemical parameters of selected Virulence, detoxification, adaptation category proteins.	S10
9.	**Table S6:** Secondary structure analysis of selected virulent proteins of *Mtb.*	S10
10.	**Table S7:** Selected hits and their binding free energy (kcal/mol) toward multiple target proteins.	S11
11.	**Table S8:** Selected compounds and their biological properties.	S12-S13

Table S1. Minimum inhibitory concentration (MIC) of different plants extracts in various solvent fractions against *M. tuberculosis* strains.

Extract code	MIC (mg/L)			
	M. tuberculosis $H_{37}Rv$ **ATCC 27294**	***M. abscessus*** **ATCC 19977**	***M. fortuitum*** **ATCC 6841**	***M. chelonae*** **ATCC 35752**
1	>64	>64	>64	>64
2	>64	>64	>64	>64
3	**64**	>64	>64	>64
4	>64	>64	>64	>64
5	>64	>64	>64	>64
6	>64	>64	>64	>64
7	>64	>64	>64	>64
8	>64	>64	>64	>64
9	>64	>64	>64	>64
10	>64	>64	>64	>64
11	>64	>64	>64	>64
12	>64	>64	>64	>64
13	>64	>64	>64	>64
14	>64	>64	>64	>64
15	>64	>64	>64	>64
16	>64	>64	>64	>64
17	>64	>64	>64	>64
18	>64	>64	>64	>64
19	>64	>64	>64	>64
20	>64	>64	>64	>64
21	>64	>64	>64	>64
22	>64	>64	>64	>64
23	**64**	>64	>64	>64
Isoniazid	0.03	NT	NT	NT
Rifampicin	0.03	NT	NT	NT
Streptomycin	1	NT	NT	NT
Ethambutol	1	NT	NT	NT
Levofloxacin	NT	2	0.06	0.06

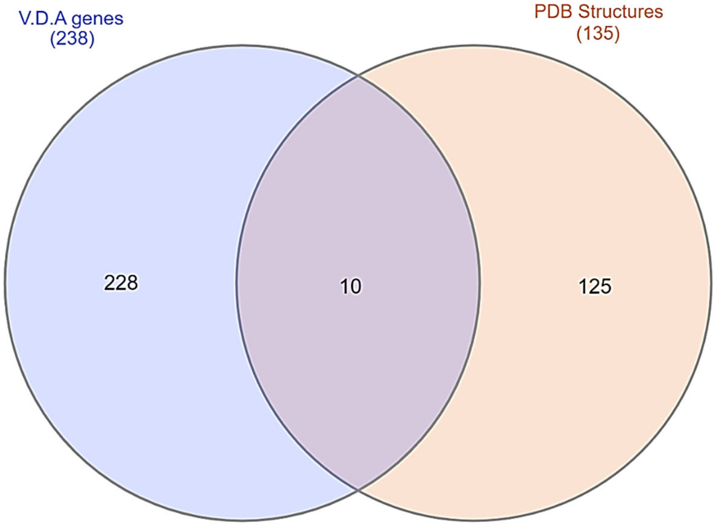

Figure S1. Venn-diagram to categorized V.D.A category and known *Mtb* PDB structure protein.

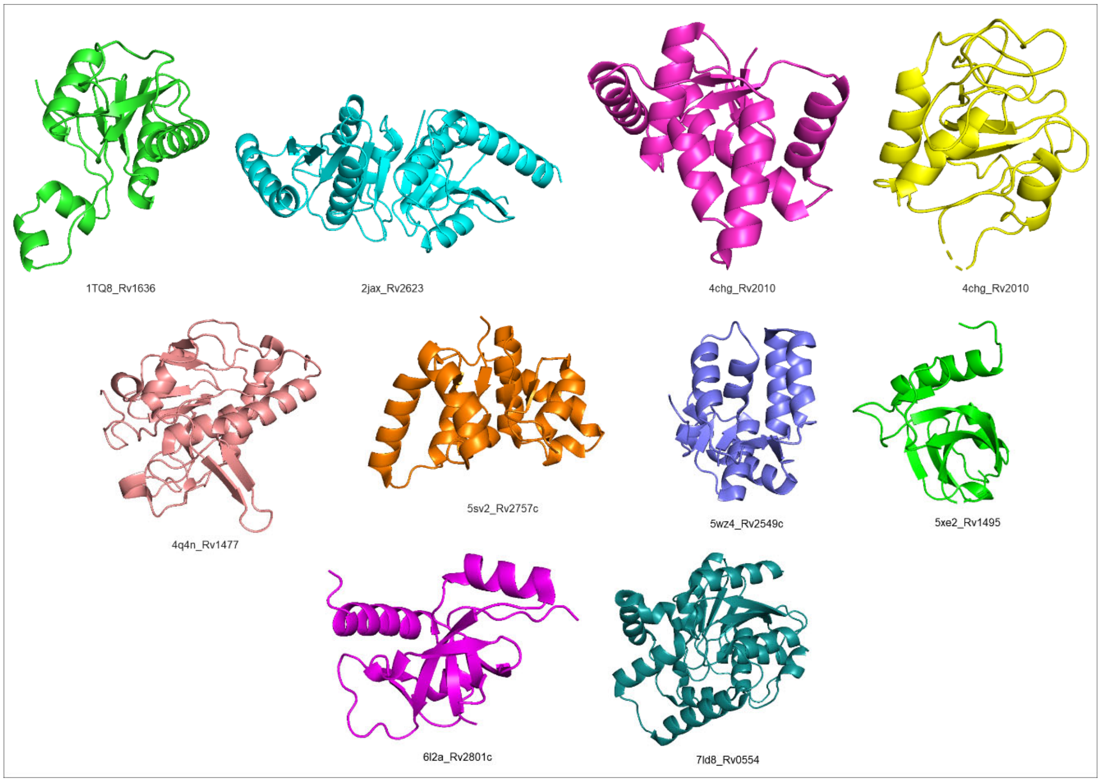

Figure S2. Crystal structure of selected *M. tuberculosis* $H_{37}Rv$ proteins (PDB: 7LD8, 4Q4N, 5XE2, 4LJ1, 1TQ8, 4CHG, 5WZ4, 2JAX, 5SV2, 6L2A).

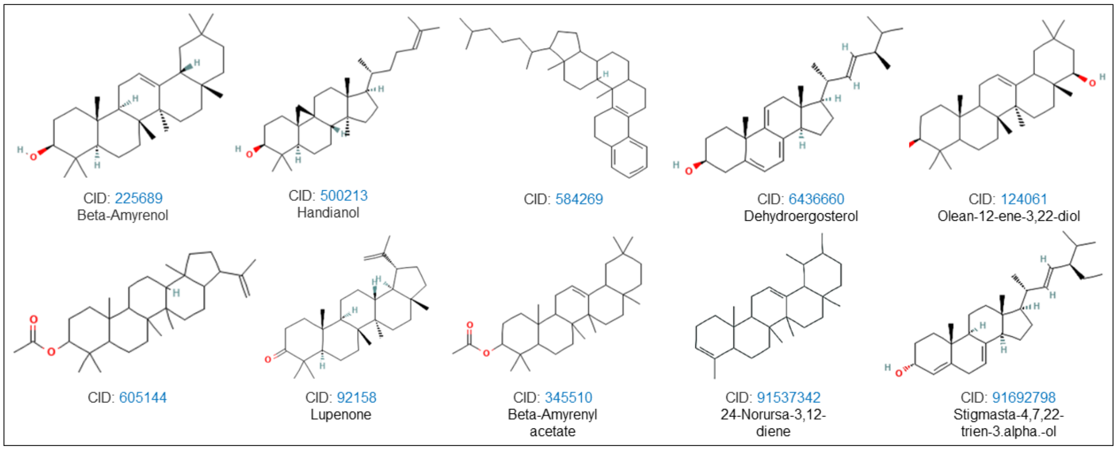

Figure S3. In multitarget proteins docking, the top hit selected phytoconstituents.

Table S2. List of the bioinformatics tools and databases for learning substantial outcome of Hypothetical protein from *M. tuberculosis* *$H_{37}Rv$*.

S.No.	Server/ Database	Application	URL
1	NCBI	Database	https://www.ncbi.nlm.nih.gov/genome/?term=h37RV
2	Mycobrowser	Sequence retrieval	https://mycobrowser.epfl.ch/genes/
3	VICMpred	Virulence factor	http://bioinfo.icgeb.res.in/virulent
4	ProtParam	Physiochemical parameters	www.web.expasy.org/protparam
5	TBpred	Localization of TB protein.	http://crdd.osdd.net/raghava/tbpred/
6	STRING	Protein-protein interaction	https://string-db.org/cgi/network.pl?taskId=BUe3enVFzh8M
7	STITCH	Protein- chemical interaction.	http://stitch1.embl.de/
8	PSIPRED	Secondary Structure	http://bioinf.cs.ucl.ac.uk
9	RAMPAGE	Structure validation	https://services.mbi.ucla.edu/SAVES/
10	ChemDraw Ultra 12.0	Draw 3D compound's structure	-
11	InstaDock Vina	Molecular docking	-
12	PyMOL	Visualizer	-
13	Discovery Studio	2D interaction	-

Table S3. Phytochemical constituents identified in the ethyl acetate aerial part extract of *A. aspera* using gas chromatography-mass spectrometry.

Peak#	R. Time	Area	Area%	Name
1	7.495	773531	0.23	1-Pentadecene
2	7.593	418354	0.12	TETRADECANE
3	7.762	585922	0.17	5-DECYNE-4,7-DIOL, 2,4,7,9-TETRAMETHYL-
4	8.280	675148	0.20	5,9-Undecadien-2-one, 6,10-dimethyl-
5	8.877	270775	0.08	PENTADECANE
6	9.089	1844602	0.54	PHENOL, 2,4-BIS(1,1-DIMETHYLETHYL)-
7	9.504	824221	0.24	2(4H)-Benzofuranone, 5,6,7,7a-tetrahydro-4,4,7a-trimethyl
8	9.838	1864425	0.55	N,N-BIS(2-HYDROXYETHYL)DODECANAMIDE
9	10.016	1749833	0.51	1-HEXADECENE
10	10.097	829629	0.24	Tetradecane
11	11.205	1007133	0.30	2-Propenoic acid, tridecyl ester
12	12.067	2950394	0.87	Tetradecanoic acid
13	12.285	1915922	0.56	1-Nonadecene
14	12.748	1116488	0.33	Neophytadiene
15	12.817	3702276	1.09	2-Pentadecanone, 6,10,14-trimethyl-
16	12.963	635774	0.19	14-Pentadecenoic acid
17	13.059	879473	0.26	1,2-Benzenedicarboxylic acid, bis(2-methylpropyl) ester
18	13.295	723814	0.21	17-PENTATRIACONTENE
19	13.558	2297475	0.68	5,9,13-Pentadecatrien-2-one, 6,10,14-trimethyl-, (E,E)-
20	13.667	6447825	1.90	HEXADECANOIC ACID, METHYL ESTER
21	13.752	613115	0.18	Benzenepropanoic acid, 3,5-bis(1,1-di-methylethyl)-4-hydr
22	13.883	237926	0.07	1-HEXADECEN-3-OL, 3,5,11,15-TETRAMETHYL-
23	13.950	910874	0.27	9-HEXADECENOIC ACID
24	14.203	50955888	14.98	n-Hexadecanoic acid
25	14.337	2338050	0.69	9-OCTADECENOIC ACID (Z)-
26	15.100	1203664	0.35	HEPTADECANOIC ACID
27	15.307	6156436	1.81	9,12-Octadecadienoic acid (Z,Z)-, methyl ester
28	15.366	5668991	1.67	9-Octadecenoic acid (Z)-, methyl ester
29	15.491	16397172	4.82	Phytol
30	15.601	789766	0.23	Methyl stearate
31	15.823	18021416	5.30	9,12-OCTADECADIENOIC ACID (Z,Z)-
32	15.872	19828608	5.83	cis-9-Hexadecenal
33	16.050	2244457	0.66	Octadecanoic acid
34	16.208	1188140	0.35	9-TRICOSENE, (Z)-
35	16.378	2138708	0.63	Phytol, acetate
36	17.127	565804	0.17	2-Methyltetracosane
37	17.372	1109140	0.33	Methyl 18-methylnonadecanoate
38	17.550	372972	0.11	Cyclohexanecarboxamide, N-(4-fluorophenyl)-
39	17.610	2172539	0.64	4,8,12,16-Tetramethylheptadecan-4-olide
40	17.786	2264224	0.67	FARNESYL ACETONE A
41	18.249	713268	0.21	Eicosanal-
42	18.793	3251974	0.96	n-Tetracosanol-1
43	19.503	430100	0.13	Docosanoic acid, ethyl ester
44	19.621	853915	0.25	Octacosyl acetate
45	19.698	1020727	0.30	Eicosanoic acid, 2-(acetyloxy)-1-[(acetyloxy)me-thyl]ethyl e
46	20.283	267266	0.08	Octadecane, 1-iodo-
47	20.350	1128598	0.33	1-Heptacosanol

48	20.521	490544	0.14	Tetracosanoic acid, methyl ester
49	20.652	419992	0.12	(E)-Hexadec-2-enal
50	20.979	2537308	0.75	13-Docosenamide, (Z)-
51	21.121	14310068	4.21	Squalene
52	21.309	769003	0.23	1,6,10,14,18,22-Tetracosahexaen-3-ol, 2,6,10,15,19,23-hex
53	21.383	2040655	0.60	.alpha.-Tocospiro A
54	21.548	2571707	0.76	.alpha.-Tocospiro B
55	21.863	263618	0.08	Disulfide, didodecyl
56	21.952	1962881	0.58	Oxirane, 2,2-dimethyl-3-(3,7,12,16,20-pentamethyl-3,7,11,
57	22.496	963942	0.28	Ergosta-5,7,9(11),22-tetraen-3-ol, (3.beta.,22E)-
58	22.682	698890	0.21	ERGOSTA-5,22-DIEN-3-OL, (3.BETA.,22E,24S)-
59	22.870	346416	0.10	Hexadecanamide
60	22.935	1460955	0.43	Stigmasta-4,7,22-trien-3.alpha.-ol
61	23.083	275604	0.08	.beta.-Tocopherol
62	23.140	381403	0.11	Stigmasta-5,22-dien-3-ol, acetate, (3.beta.)-
63	23.261	1712582	0.50	.gamma.-Tocopherol
64	23.551	2979708	0.88	Stigmasta-5,22-dien-3-ol, acetate, (3.beta.)-
65	23.777	1839492	0.54	STIGMAST-5-EN-3-OL, OLEAT
66	24.098	5933162	1.74	Vitamin E
67	24.683	1107733	0.33	2H-1-BENZOPYRAN, 3,4,4A,5,6,8A-HEXAHY-DRO-2,5,
68	24.925	1546130	0.45	Lathosterol
69	25.202	2926186	0.86	SOLANESOL
70	25.553	5053865	1.49	Ergost-5-en-3-ol, (3.beta.)-
71	25.917	24590581	7.23	Stigmasterol
72	26.118	7853233	2.31	24-NOR-CHOLEST-22-ENE
73	26.465	2377824	0.70	Ergost-7-en-3-ol
74	26.642	1570747	0.46	22-Stigmasten-3-one
75	26.863	29419710	8.65	.gamma.-Sitosterol
76	27.252	1661821	0.49	7,22-Ergostadienone
77	27.616	7664879	2.25	.beta.-Amyrin
78	27.904	11019318	3.24	Stigmast-7-en-3-ol, (3.beta.,5.alpha.,24S)-
79	28.254	3305541	0.97	9,19-Cyclolanost-24-en-3-ol, (3.beta.)-
80	28.882	2486691	0.73	5-(1,5-DIMETHYLHEXYL)-5A,7B-DIMETHYL-2,2A,2B
81	29.171	2609211	0.77	STIGMAST-4-EN-3-ONE
82	29.373	8470333	2.49	9,19-Cyclolanostan-3-ol, 24-methylene-, (3.beta.)-
83	30.069	1639645	0.48	Lup-20(29)-en-3-ol, acetate, (3.beta.)-
84	31.410	2840849	0.84	Phytyl palmitate
85	32.388	1838788	0.54	LUP-20(29)-EN-3-YL ACETATE
86	33.904	1222814	0.36	SOLANESOL
87	37.110	2674619	0.79	Isopropyl linoleate

Table S4. Phytochemical constituents identified in the ethyl acetate flower ash extract of *C. gigantea* using gas chromatography-mass spectrometry.

Peak#	R. Time	Area	Area%	Name
1	10.015	1049325	0.07	1-Pentadecene
2	12.063	2541556	0.17	Tetradecanoic acid
3	12.815	817504	0.06	2-Pentadecanone, 6,10,14-trimethyl-
4	13.119	4226712	0.29	Pentadecanoic acid
5	13.669	21459140	1.48	HEXADECANOIC ACID, METHYL ESTER
6	13.957	2950312	0.20	9-Octadecen-1-ol, (Z)-
7	14.248	55237880	3.80	n-Hexadecanoic acid
8	14.333	3879939	0.27	HEXADECANOIC ACID, ETHYL ESTER
9	15.043	1025451	0.07	4-Oxazolecarboxylic acid, 4,5-dihydro-2-phenyl-, 1-methy
10	15.137	2259099	0.16	KAUR-16-ENE
11	15.312	24787855	1.71	9,12-Octadecadienoic acid (Z,Z)-, methyl ester
12	15.373	27535750	1.90	9-Octadecenoic acid, methyl ester, (E)-
13	15.502	1301829	0.09	Phytol
14	15.602	3624595	0.25	Methyl stearate
15	15.917	55470106	3.82	9,12-Octadecadienoic acid (Z,Z)-
16	16.096	2608913	0.18	9-OCTADECENOIC ACID (Z)-
17	16.259	2082406	0.14	Octadecanamide
18	17.128	6313870	0.43	Heneicosane
19	17.377	2753535	0.19	Methyl 18-methylnonadecanoate
20	17.566	3965414	0.27	CYCLOHEXANE, DECYL-
21	17.786	2191892	0.15	9-Octadecenamide, (Z)-
22	17.962	3686080	0.25	Tetratetracontane
23	18.575	1238454	0.09	17-Pentatriacontene
24	18.771	8418747	0.58	Heneicosane
25	19.012	1300090	0.09	DOCOSANOIC ACID, METHYL ESTER
26	19.449	1402223	0.10	Hexadecanoic acid, phenylmethyl ester
27	19.697	3563535	0.25	Eicosanoic acid, 2-(acetyloxy)-1-[(acetyloxy)methyl] ethyl
28	19.774	3189240	0.22	2-OCTADECENOIC ACID, METHYL ESTER
29	20.117	2665701	0.18	Eicosyl trifluoroacetate
30	20.290	5652738	0.39	Heneicosane
31	20.524	1203777	0.08	Tetracosanoic acid, methyl ester
32	20.781	2622561	0.18	9,12-Octadecadienoic acid (Z,Z)-, phenylmethyl ester
33	20.979	9660544	0.66	9-OCTADECENAMIDE
34	21.121	4385069	0.30	Squalene
35	21.384	1189944	0.08	. alpha. -Tocospiro B
36	21.585	6493662	0.45	1-EICOSANOL
37	21.759	3097061	0.21	Tetracontane
38	21.953	1398822	0.10	Oxirane, 2,2-dimethyl-3-(3,7,12,16,20-pentamethyl-3,7,11,
39	22.037	2408085	0.17	METHYL DIHYDROMALVALATE
40	22.935	3298080	0.23	Stigmasta-4,7,22-trien-3.alpha.-ol
41	23.259	1827051	0.13	.gamma.-Tocopherol
42	23.397	3123229	0.21	1-EICOSANOL
43	23.483	762688	0.05	1-EICOSANOL
44	23.553	1738051	0.12	Stigmasta-5,22-dien-3-ol, acetate, (3.beta.)-
45	23.632	1471295	0.10	9-(3,3-DIMETHYL-2-OXIRANYL)-2,7-DIMETHYL-2,6-
46	23.779	2467991	0.17	STIGMAST-5-EN-3-OL, (3.BETA.)-
47	24.104	2032042	0.14	Vitamin E

48	24.729	1338461	0.09	.ALPHA.-SELINENE
49	25.574	36839194	2.54	ERGOST-5-EN-3-OL, (3.BETA.,24R)-
50	25.951	57165303	3.93	Stigmasterol
51	26.923	56259699	3.87	.gamma.-Sitosterol
52	27.180	2789530	0.19	Fucosterol
53	27.664	57904082	3.99	.beta.-Amyrin
54	27.883	6193541	0.43	24-Norursa-3,12-diene
55	28.054	8952706	0.62	4,22-Stigmastadiene-3-one
56	28.332	3373817	0.23	9,19-Cyclolanost-24-en-3-ol, (3.beta.)-
57	28.538	83296093	5.73	METHYL COMMATE D
58	28.990	1963487	0.14	Stigmasta-3,5-diene
59	29.318	199402288	13.72	4,4,6a,6b,8a,11,11,14b-Octamethyl-1,4,4a,5,6,6a,6b,7,8,8a,
60	29.621	3253155	0.22	METHYL COMMATE B
61	29.852	4570682	0.31	Lup-20(29)-en-3-one
62	30.361	286766478	19.74	Lup-20(29)-en-3-ol, acetate, (3.beta.)-
63	30.697	79643969	5.48	Lupeol
64	31.478	16540535	1.14	Cyclopropa[5,6]stigmast-22-en-3-ol, 3',6-dihydro-, (3.beta.,
65	32.245	44151018	3.04	A'-Neogammacer-22(29)-en-3-ol, acetate, (3.beta.,21.beta.)
66	32.630	100754447	6.93	LUP-20(29)-EN-3-YL ACETATE
67	36.603	41854800	2.88	Olean-12-en-3-ol, acetate, (3.beta.)-
68	38.355	51580680	3.55	METHYL COMMATE A
		1452973808	100.00	

Table S5. Physiochemical parameters of selected Virulence, detoxification, adaptation category proteins.

S. No.	Protein Seq.	No. of amino acids	MW (~kDa)	pI	Ext. Cof.	Instability index	Aliphatic index	GRAVY
1	Rv0554	262	28.349	5.26	27055	27.17 (Stable)	89.08	0.065
2	Rv1477	472	49.808	9.08	46995	41.76 (Unstable)	79.34	-0.251
3	Rv1495	105	11.377	8.09	22460	22.40 (Stable)	91.05	-0.190
4	Rv1566c	230	23.964	10.23	15930	55.24 (Unstable)	80.13	-0.111
5	Rv1636	146	15.312	5.51	5960	25.53 (Stable)	106.30	0.005
6	Rv2010	132	14.731	6.29	14105	55.56 (Unstable)	106.52	0.061
7	Rv2549c	131	14.619	6.51	30480	17.96 (Stable)	79.01	-0.137
8	Rv2623	297	31.652	5.45	47105	52.60 (Unstable)	105.32	0.104
9	Rv2757c	138	15.773	6.65	23950	32.09 (Stable)	91.23	-0.307
10	Rv2801c	118	12.858	9.10	12490	34.20 (Stable)	107.46	-0.067

Table S6. Secondary structure analysis of selected virulent proteins of *Mtb.*

S.no.	Protein	Alpha helix	Extended strand	Beta turn	Random coil
1	Rv0554	117 (44.66%)	37 (14.12%)	14 (5.34%)	94 (35.88%)
2	Rv1477	230 (48.73%)	50 (10.59%)	28 (5.93%)	164 (34.75%)
3	Rv1495	35 (33.33%)	25 (23.81%)	2 (1.90%)	43 (40.95%)
4	Rv1566c	35 (15.22%)	37 (16.09%)	27 (11.74%)	131 (56.96%)
5	Rv1636	68 (46.58%)	28 (19.18%)	8 (5.48%)	42 (28.77%)
6	Rv2010	78 (59.09%)	14 (10.61%)	6 (4.55%)	34 (25.76%)
7	Rv2549c	72 (54.96%)	23 (17.56%)	8 (6.11%)	28 (21.37%)
8	Rv2623	115 (38.72%)	58 (19.53%)	14 (4.71%)	110 (37.04%)
9	Rv2757c	87 (63.04%)	13 (9.42%)	9 (6.52%)	29 (21.01%)
10	Rv2801c	44 (37.29%)	23 (19.49%)	8 (6.78%)	43 (36.44%)

Table S7. Selected hits and their binding free energy (kcal/mol) toward multiple target proteins.

S. No.	Receptor	Name of the ligand	Binding Free Energy (kcal/mol)	pKi	Ligand Efficiency (kcal/mol/non-H atom)	Torsional Energy
1	Rv1636_A*	225689	-10.6	7.77	0.3118	0.3113
2	Rv1636_G*	225689	-10.6	7.77	0.3118	0.3113
3	Rv2623_A*	500213	-8.8	6.45	0.2316	1.5565
4	Rv2623_G*	500213	-9.2	6.75	0.2421	1.5565
5	Rv2010_A*	500213	-7.5	5.5	0.1974	1.5565
6	Rv2010_G*	124061	-8.1	5.94	0.225	0.6226
7	Rv1566_A*	225689	-10.7	7.85	0.3147	0.3113
8	Rv1566_G*	225689	-10.6	7.77	0.3118	0.3113
9	Rv1477_A*	225689	-7.8	5.72	0.2294	0.3113
10	Rv1477_G*	605144	-8.2	6.01	0.2103	0.9339
11	Rv2757c_A*	584269	-9	6.6	0.2143	1.5565
12	Rv2757c_G*	605144	-9.1	6.67	0.2333	0.9339
13	Rv2549c_A*	225689	-7.9	5.79	0.2324	0.3113
14	Rv2549c_G*	92158	-8	5.87	0.2353	0.3113
15	Rv1495_A*	225689	-8.7	6.38	0.2559	0.3113
16	Rv1495_G*	345510	-8.8	6.45	0.2316	0.6226
17	Rv2801c_A*	6436660	-7.9	5.79	0.2194	1.5565
18	Rv2801c_G*	91735342	-7.9	5.79	0.2548	0
19	Rv0554_A*	6436660	-9.6	7.04	0.2667	1.5565
20	Rv0554_G*	91692798	-9.7	7.11	0.2553	1.8678

A* Top hit selected from GCMS studies of *Achyranthes aspera* (medicinal plant).
G* Top hit selected from GCMS studies of *Calotropis gigantea* (medicinal plant).

Table S8. Selected compounds and their biological properties.

S.No.	PubChem ID	Pa	Pi	Biological activity
1	225689	0,977	0,001	Insulin promoter
		0,976	0,002	Caspase 3 stimulant
		0,944	0,001	Transcription factor stimulant
		0,944	0,001	Transcription factor NF kappa B stimulant
		0,939	0,004	Mucomembranous protector
		0,926	0,002	Hepatoprotectant
		0,923	0,004	Apoptosis agonist
		0,916	0,005	Antineoplastic
		0,913	0,002	Oxidoreductase inhibitor
		0,909	0,002	Membrane integrity antagonist
		0,903	0,002	Chemopreventive
2	500213	0,964	0,003	Hypolipemic
		0,952	0,002	Chemopreventive
		0,947	0,002	Cholesterol antagonist
		0,933	0,000	Sterol 24-C-methyltransferase inhibitor
3	584269	0,928	0,003	Prostaglandin-E2 9-reductase inhibitor
		0,919	0,004	Alkenylglycerophosphocholine hydrolase inhibitor
4	6436660	0,959	0,002	Antihypercholesterolemic
		0,947	0,003	Caspase 3 stimulant
		0,928	0,001	Alcohol O-acetyltransferase inhibitor
		0,917	0,003	Cholesterol antagonist
		0,912	0,004	Hypolipemic
		0,910	0,002	Oxidoreductase inhibitor
5	124061	0,980	0,002	Caspase 3 stimulant
		0,965	0,002	Insulin promoter
		0,939	0,001	Transcription factor stimulant
		0,939	0,001	Transcription factor NF kappa B stimulant
		0,934	0,002	Hepatoprotectant
		0,914	0,005	Antineoplastic
		0,909	0,004	Apoptosis agonist
		0,904	0,002	Oxidoreductase inhibitor
		0,902	0,006	Testosterone 17beta-dehydrogenase (NADP+) inhibitor
6	605144	0,942	0,004	Antineoplastic
		0,929	0,003	Antiprotozoal (Leishmania)
		0,923	0,003	Caspase 3 stimulant
		0,912	0,001	Transcription factor NF kappa B stimulant
		0,912	0,001	Transcription factor stimulant
		0,906	0,002	Hepatoprotectant
7	92158	0,955	0,004	Antineoplastic
		0,947	0,001	Transcription factor NF kappa B stimulant
		0,947	0,001	Transcription factor stimulant
		0,921	0,003	Antiprotozoal (Leishmania)
		0,903	0,004	Apoptosis agonist
		0,955	0,004	Antineoplastic
8	345510	0,964	0,002	Lipid metabolism regulator
		0,960	0,003	Mucomembranous protector
		0,946	0,003	Caspase 3 stimulant
		0,941	0,002	Insulin promoter
		0,935	0,002	Hepatoprotectant
		0,925	0,001	Oxidoreductase inhibitor
		0,922	0,005	Antineoplastic
		0,918	0,002	Membrane integrity antagonist

		0,915	0,001	Transcription factor stimulant
		0,915	0,001	Transcription factor NF kappa B stimulant
		0,915	0,002	Chemopreventive
		0,911	0,004	Apoptosis agonist
9	91537342	0,911	0,004	Apoptosis agonist
		0,907	0,005	Antineoplastic
		0,900	0,004	Antiinflammatory
		0,827	0,002	Nitric oxide antagonist
		0,812	0,004	Hepatic disorders treatment
		0,803	0,003	Transcription factor NF kappa B stimulant
		0,803	0,003	Transcription factor stimulant
		0,789	0,005	Antiprotozoal (Leishmania)
		0,771	0,005	Antileukemic
		0,755	0,005	Hepatoprotectant
10	91692798	0,976	0,001	Antihypercholesterolemic
		0,961	0,001	Cholesterol antagonist
		0,920	0,004	Hypolipemic
		0,900	0,001	Alcohol O-acetyltransferase inhibitor
		0,900	0,002	Oxidoreductase inhibitor
